# Self‐Induced Phase Locking of Terahertz Frequency Combs in a Phase‐Sensitive Hyperspectral Near‐Field Nanoscope

**DOI:** 10.1002/advs.202200410

**Published:** 2022-06-16

**Authors:** Valentino Pistore, Eva Arianna Aurelia Pogna, Leonardo Viti, Lianhe Li, A. Giles Davies, Edmund H. Linfield, Miriam Serena Vitiello

**Affiliations:** ^1^ NEST CNR‐Istituto Nanoscienze and Scuola Normale Superiore Pisa 5612 Italy; ^2^ School of Electronic and Electrical Engineering University of Leeds Leeds LS2 9JT UK

**Keywords:** near‐field nanoscopy, terahertz, hyperspectral nanoimaging, frequency combs

## Abstract

Chip‐scale, electrically‐pumped terahertz (THz) frequency‐combs (FCs) rely on nonlinear four‐wave‐mixing processes, and have a nontrivial phase relationship between the evenly spaced set of emitted modes. Simultaneous monitoring and manipulation of the intermode phase coherence, without any external seeding or active modulation, is a very demanding task for which there has hitherto been no technological solution. Here, a self‐mixing intermode‐beatnote spectroscopy system is demonstrated, based on THz quantum cascade laser FCs, in which light is back‐scattered from the tip of a scanning near‐field optical‐microscope (SNOM) and the intracavity reinjection monitored. This enables to exploit the sensitivity of FC phase‐coherence to optical feedback and, for the first time, manipulate the amplitude, linewidth and frequency of the intermode THz FC beatnote using the feedback itself. Stable phase‐locked regimes are used to construct a FC‐based hyperspectral, THz s‐SNOM nanoscope. This nanoscope provides 160 nm spatial resolution, coherent detection of multiple phase‐locked modes, and mapping of the THz optical response of nanoscale materials up to 3.5 THz, with noise‐equivalent‐power (NEP) ≈400 pW √Hz^−1^. This technique can be applied to the entire infrared range, opening up a new approach to hyper‐spectral near‐field imaging with wide‐scale applications in the study of plasmonics and quantum science, inter alia.

## Graphical abstract

Hyperspectral terahertz near field optical microscope comprising a miniaturized quantum cascade laser frequency comb, acting simultaneously as a source and as a detector. Quantum nanomaterials and related collective mode excitations can be probed at the nanoscale.

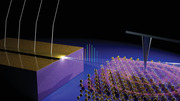

## 1 Introduction

Frequency combs (FCs) have revolutionized the fields of optical synthesis,^[^ ^1^ ^]^ metrology,^[^ ^2^ ^]^ and quantum science,^[^ ^3^ ^]^ with an ever‐growing range of applications in a plethora of distinctive fields including molecular spectroscopy,^[^ ^4^ ^]^ telecommunications,^[^ ^5^ ^]^ and imaging.^[^ ^6^ ^]^ They can be generated by several well‐established techniques, most notably through the stabilization of the repetition rate and carrier envelope phase of mode‐locked lasers.^[^ ^7^ ^]^ However, such approaches cannot be easily translated to the low‐energy regions of the electromagnetic spectrum, i.e. across the terahertz (THz) and millimeter ranges, since the physics behind the operation of sources and optical components is substantially different at longer wavelengths. Alternative techniques have, therefore, been developed, including use of nonlinear crystals,^[^ ^8^ ^]^ optical parametric oscillators,^[^ ^9^ ^]^ and photoconductive switches.^[^ ^10^ ^]^ However, the widespread use of THz FCs has been hampered by the size and complexity of the associated optical apparatus.

Miniaturized quantum cascade lasers (QCLs) have emerged over the last few years as the most promising source of FCs^[^ ^11^ ^]^ in the mid‐IR and THz spectral ranges and widely exploited for dual‐comb spectroscopy^[^ ^12^, ^13^ ^]^ and hyperspectral imaging^[^ ^14^ ^]^ in the far‐field in combination with hot‐electron bolometers.^[^ ^14^ ^]^ Owing to the large third‐order nonlinearity of their gain media (*χ* ^(3)^ ≈0.9 × 10^−15^ m^2^ V^−^^2^ for mid‐infrared QCLs and *χ* ^(3)^≈0.7 × 10^−15^ m^2^ V^−^^2^ for THz QCLs),^[^ ^15^, ^16^ ^]^ these sources display spontaneous FC operation by self‐phase locking of the cavity modes through intracavity four‐wave mixing (FWM). Their chip‐scale size (which in our specific FCs are ≈50–90 µm × 3 mm × 15–17 µm), high‐power (≈10 mW) emission,^[^ ^17^ ^]^ “bandstructure‐by‐design,” broad (up to one octave) bandwidth,^[^ ^18^ ^]^ and high spectral purity (≈100 Hz intrinsic linewidths)^[^ ^19^ ^]^ are inherent characteristics which make these devices ideal for FC operation.

The observation of a single and narrow intermodal beat‐note, originating from the beating of the cavity modes of a QCL, has traditionally been considered a necessary but not sufficient condition for FC operation.^[^ ^20^ ^]^ In fact, it only shows that at least some of the modes are coherently locked, and does not provide information on whether this is true for the whole spectrum or just a smaller subgroup of modes. Two techniques have thus been developed to confirm the existence of a fixed‐phase relation between all modes in QCL‐FCs: shifted wave interference Fourier transform spectroscopy (SWIFTS)^[^ ^21^ ^]^ and Fourier analysis of comb emission (FACE).^[^ ^22^ ^]^ However, they both require sophisticated experimental arrangements and have limitations: the FACE technique needs sufficiently high power‐per‐mode to retrieve the phases of the modes,^[^ ^23^ ^]^ while SWIFTS requires a mechanical scan and cannot access the relative offset frequency of noncontinuous portions of combs.^[^ ^24^ ^]^

An alternative technique is self‐mixing intermode beatnote (SMIB) spectroscopy, which exploits the beatnote sensitivity to external optical feedback (EOF) and uses this to verify the locking of the modes. Such a technique has been exploited in the mid‐IR to control the intermode beatnote of QCL combs by means of microelectromechanical drives.^[^ ^25^ ^]^ At THz frequencies, a recent work^[^ ^26^ ^]^ has shown that the moving mirror of a Fourier transform infrared spectroscopy (FTIR) system provides feedback and the frequency shift of the beatnote can be measured against mirror position for a THz QCL with a single‐plasmon waveguide. The resulting interferogram provided information on the THz modes contributing to the beatnote, and was in agreement with the predictions of the Lang–Kobayashi equations.^[^ ^27^ ^]^ The same method was also employed to assess the comb operation of THz QCLs with double‐metal waveguides.^[^ ^28^, ^29^ ^]^ However, no study has so far been undertaken to address the link between the beatnote response to feedback and the terminal voltage variation induced by self‐mixing in a THz QCL lasing cavity,^[^ ^30^, ^31^ ^]^ which has been largely exploited for sensing and imaging applications.^[^ ^32^, ^33^ ^]^ Here, we demonstrate the first‐ever hyperspectral scattering‐type scanning near‐field optical microscope (s‐SNOM) imaging system at frequencies in the 2.29–3.60 THz range, relying on a 6 mW excitation source based on a comb‐emitting THz QCL and propose a new approach to SMIB spectroscopy. By coupling the THz QCL FC to the tip of the s‐SNOM we investigate the sensitivity of the beatnote to optical feedback and its relation to the complex self‐mixing (voltage) response generated by THz QCL FCs. We show that the intermode FC beatnote is modulated in intensity, position, and linewidth by the feedback, being sensitive to the feedback phase, and can be used to unambiguously identify the phase‐locked modes of the FC. The feedback itself then drives the laser in stable comb regime, impossible to be accessible spontaneously. Our coherent FC nanoscope provides nanoscale resolution suitable for probing the optical response of materials, molecules, and bio‐samples, at multiple THz frequencies, simultaneously.

During the last decade, THz nanoscopy has attracted great interest due to its potential for nanoscale imaging of material and device properties.^[^ ^34^, ^35^, ^36^, ^37^ ^]^ So far, THz‐SNOM imaging has been performed using either broadband sources, such as those used in time‐domain spectroscopy (TDS) systems that show a sever performance drop at frequencies > 1.5 THz,^[^ ^38^ ^]^ or gas lasers,^[^ ^34^ ^]^ or bulky (not table‐top) and high‐cost free electron lasers,^[^ ^39^ ^]^ or single‐mode electronic sources,^[^ ^40^ ^]^ or by using THz QCLs in a phase‐sensitive detectorless configuration.^[^ ^36^, ^41^, ^42^, ^43^, ^44^, ^45^, ^46^ ^]^ The main disadvantages of broadband THz sources are their cumbersome setups, which require an external laser to generate ultrashort pump pulses, their limited (µW) output‐powers and the need of sensitive THz detectors,^[^ ^47^, ^48^ ^]^ while electronic sources and gas lasers only operate on specific modes, and are usually neither compact nor high power. In contrast, when single‐mode THz QCLs are used for THz nanoscopy, they are powerful, do not require a separate detector, and can be operated in a multifrequency configuration, albeit with randomly emitted modes.^[^ ^45^ ^]^ However, they cannot provide the dense, continuous, and broad spectral coverage required for hyperspectral nanoimaging applications—this is enabled by the FC‐based phase‐sensitive THz near‐field optical nanoscope demonstrated here.

## 2 Results

**Figure 1:**
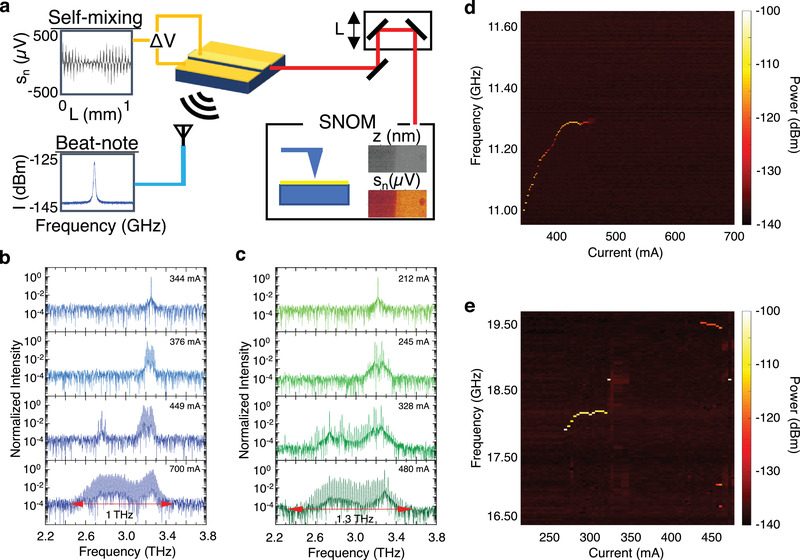
Optical feedback interferometry with THz QCL FCs: a) Schematic of the experimental apparatus: a THz QCL FC is operated in CW, and its emission is collimated by two parabolic mirrors and passed through a delay line to the s‐SNOM for inspecting the topography *z* and the THz‐near field scattering *V*_n_ of a nanosize sample, where *n* is the order of the scattering harmonics. The radiation scattered by the tip is coupled back into the QCL cavity where it generates a self‐mixing signal in the form of contact voltage *Δ* *V* oscillations. The beatnote spectrum is acquired through an optical antenna connected to a spectrum analyzer, which is placed near the laser source. The phase of the feedback is controlled by an optical delay line including a motorized stage which varies the optical path by *L*. b,c) Normalized FTIR emission spectra of b) FC_1_ and c) FC_2_ at different driving currents. d,e) Beatnote maps of d) FC_1_ and e) FC_2_ without feedback.

A schematic of the experimental apparatus is shown in **Figure** **1**a. A THz QCL FC, fabricated in a gold–gold double‐metal waveguide (see the Experimental Section), is driven in continuous‐wave (CW) at a fixed temperature of 23 K. The radiation backscattered by the s‐SNOM atomic force microscopy (AFM) tip, operated in tapping mode, is reinjected into the laser cavity and the self‐mixing signal is detected as a perturbation Δ*V* to the QCL voltage. Simultaneously, a wire‐based optical antenna placed in proximity to the cryostat is used to measure the intermode beatnote signal radiated in the free space by the THz QCL FC (see the Experimental Section). An optical delay‐line is also incorporated into the apparatus to vary the optical path, and control the phase of the optical feedback (see the Experimental Section). In the weak optical feedback regime, here guaranteed by the use of an AFM tip as scattering element (giving a feedback parameter *C* < 0.1 see ref. [49]), theoretical studies^[^ ^50^ ^]^ have demonstrated that the self‐mixing response of a broadband THz QCL (having linewidth enhancement factor^[^ ^19^, ^51^ ^]^ *ɑ* ≈0.4 and gain bandwidth^[^ ^52^, ^53^ ^]^ >1.3 THz) is proportional to the feedback strength, thus ensuring the applicability of our technique to spectroscopic studies.

To validate our technique, we used two prototypical THz QCL FCs (see the Experimental Section), named FC_1_ and FC_2_ henceforth. Details on the FACE experiments performed to assess their fully phase‐stabilized nature are given.^[^ ^12^, ^53^ ^]^ Their heterogeneous active regions are designed to cover a broad spectral range (1.0 THz for FC_1_ and 1.3 THz for FC_2_), centered at 3 THz (Figure 1b,c). However, they show quite distinct beatnote maps (Figure 1d,e). At low driving current, and no feedback from the AFM tip, FC_1_ displays a single and narrow beatnote (linewidth 3.8–5.0 kHz)^[^ ^54^ ^]^ over a relatively broad range of driving current (340–460 mA) (Figure 1d). At higher currents, the beatnote then broadens and disappears.^[^ ^54^ ^]^ The presence of a single and narrow linewidth (from hundreds of Hz up to few tens of kHz) beatnote is generally assumed to be a genuine indication of comb operation, i.e. of the emission of a set of phase‐locked modes. On the contrary, large beatnotes (with linewidths >100 kHz) can be due to the beating of uncorrelated modes with thermally fluctuating frequencies and phase. FC_2_ (Figure 1e) shows a much more complex behavior with a single and narrow (linewidth 27–90 kHz) beatnote (Figure S5, Supporting Information) extending over a 58 mA range of current (270–330 mA) followed by a regime in which the beatnote disappears. Multiple beatnotes, spreading over a 2.5 GHz range, are then observed at higher biases (435–460 mA current range). This behavior reflects the splitting of the emission spectrum into several subcombs,^[^ ^55^ ^]^ each with its own mode‐spacing, carrier‐envelope frequency and specific response to bias variation. Finally, a single and narrow (linewidth 27 kHz) beatnote appears over a small current range (480–490 mA), where the laser spectrum is spectrally rich.

**Figure 2:**
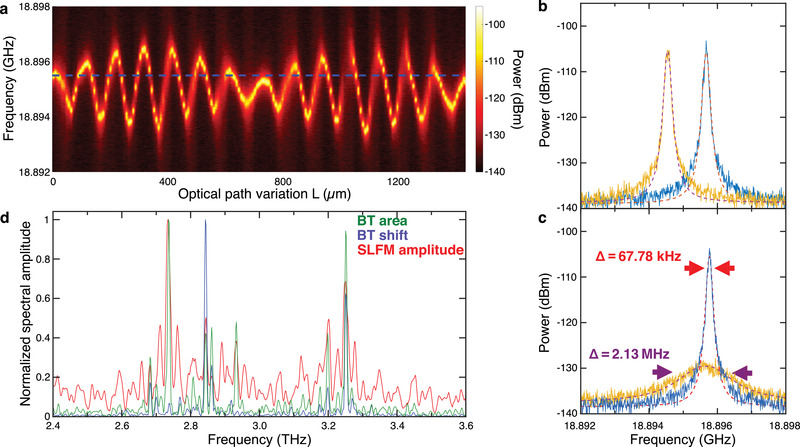
Phase‐dependent intermode beatnote sensitivity to optical feedback: a) Beatnote map as a function of the optical path length (*L*) of FC_2_ driven in the single beatnote regime at 328 mA, with feedback generated by allowing the s‐SNOM tip to approach a gold marker. The frequency position of the beatnote without the feedback is indicated by the blue dashed line. b) Intermode beatnote of FC_2_ driven at 328 mA with feedback for *L* = 1224 µm (blue line) and *L* = 72 µm (orange line). The data are fit to Lorentzian functions to determine the areas under the peaks, their full widths at half maximum (FWHMs) and their central position (red dashed lines). c) Intermode beatnote of FC_2_ driven at 328 mA with feedback for *L* = 1224 µm (blue line) and *L* = 1288 µm (orange line), and fit to Lorentzian functions (red dashed lines) from which we retrieve a reduction by ≈31 times of the FWHM (*Δ* = 67.78 kHz (red arrows), compared to *Δ* = 2.13 MHz (purple arrows)). d) Normalized Fourier spectra of the oscillations of the beatnote central frequency (blue line) and area (green line) as extracted from the analysis of the data in a) and Figure S2 (Supporting Information), together with the spectrum of the self‐mixing signal (red line).

Interestingly, when the feedback from the AFM tip is enabled, the beatnote maps of both lasers vary, with the main differences being the beatnote intensity, its linewidth and the central frequency (see **Figure** **2**; and Figure S1, Supporting Information). This means that the feedback affects the phase stability of the modes involved in the phase locking process. To understand the effect of the feedback on the beatnote signal (Section 1, Supporting Information), we operated the AFM tip in tapping mode with a 200 nm amplitude, and approached a 45 nm thick film of gold evaporated onto an undoped SiO_2_/Si substrate, exploiting the surface's high reflectivity and flat optical response in the probed spectral range. In this configuration, the tip dithering modulates the amplitude of the self‐mixing signal, without affecting its phase. We then monitored the beatnote of FC_2_, while varying the phase of the feedback from the AFM tip, by moving the delay line over a distance of 8 cm, with individual steps of 8 µm, noting the beatnote spectrum and the self‐mixing signal. An expanded section of the beatnote map as a function of *L* over a 1.5 mm range is shown in Figure 2a, while the full 8 cm range is reported in Figure S2 (Supporting Information).

The central frequency, area and full width at half maximum (FWHM) parameters of each beatnote were extracted from a Lorentzian curve fitting, with the area under the Lorentzian fit corresponding to the total power of the beatnote. When FC_2_ is driven CW at 328 mA, without feedback, the beatnote spectrum consists of a single, narrow (≈50 kHz linewidth) beatnote, centered at ≈18.895 GHz. When optical feedback is introduced, we observe a maximum shift of ≈1.5 MHz in the beatnote central frequency (Figure 2b), in agreement with previous reports.^[^ ^26^, ^28^, ^29^ ^]^ However, we also observe a sizable variation in the beatnote FWHM and, importantly, the total beatnote power (Figure 2c), with a complex dependency on the degree of feedback (Figure 2a).

The frequency shift and the variation of the beatnote FWHM were further evaluated by Fourier transforming the oscillations in the beatnote area and central frequency with respect to *L*, over its whole 8 cm range (Figure 2d). The retrieved spectra are compared with the spectrum of the self‐mixing signal, simultaneously recorded at each delay line position (Figure S3, Supporting Information). The beatnote power variation follows quite well the self‐mixing signal, with main modes located at 2.73 and 3.25 THz, and very similar amplitude distributions in the Fourier components. On the other hand, the beatnote shift (blue trace, Figure 2d), that represents the frequency spacing of the FC modes, has fewer oscillatory components, lacking modes at 2.73, 2.92, and 3.20 THz, and is dominated by the component at 2.84 THz. It thus appears that the modes driving the beatnote frequency shift are not necessarily the same as those that dominate the self‐mixing signal. This could be related to the heterogeneous architecture of the active region of this THz QCL FC, as the active regions lasing at this bias produce a single comb with two distinct bands.^[^ ^56^ ^]^ The comparison of the FTIR and beatnote shift spectra in logarithmic scale (Figure S4, Supporting Information), show that, despite the naturally higher noise floor in the beatnote‐shift spectrum, the bandwidth of the two spectra is the same. Our experiments also show that the amplitude of the self‐mixing signal is significantly influenced by the coherence state of the laser, with the highest signal‐to‐noise ratio (SNR) corresponding to having a single powerful beatnote. By tuning the feedback phase of FC_1_ with our technique, we show that comb emission can be induced in regimes with poor mode coherence (Figure S7, Supporting Information), thus extending the comb regime to spontaneously inaccessible bias ranges. This could enable comb formation at high currents where the spectral bandwidth can reach one octave in THz QCLs.^[^ ^18^ ^]^

**Figure 3:**
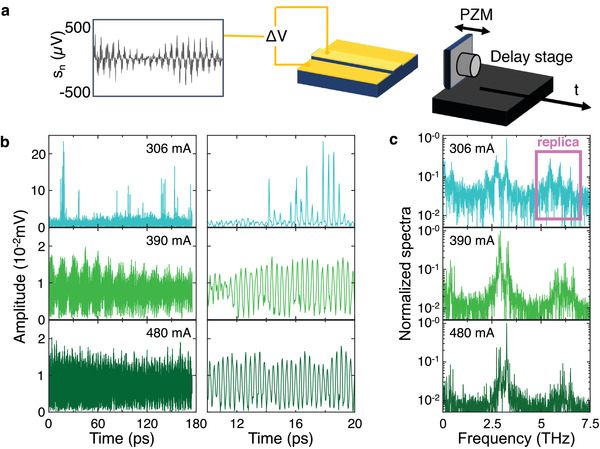
Multimode self‐mixing signal of a THz‐QCL FC: a) Schematic representation of the modified apparatus used to measure the self‐mixing response of FC_2_ with a piezoelectric mirror (PZM) injecting the feedback radiation into the laser cavity. b) Self‐mixing signal amplitude as a function of the time delay *t* = *L*/*c*, where *L* is the scan length of the optical delay line and *c* is the speed of light. Data are shown when FC_2_ is driven CW at 306, 390, and 480 mA. The right column displays expanded sections of the time‐domain traces over a 20 ps window. c) Self‐mixing signal spectra obtained by Fourier transform of time traces in b). A principal band of frequencies is observed at ≈3 THz, together with low frequency modes at tens to hundreds of GHz. The pink square highlights one of the spectral replicas that appear in self‐mixing interferometry for feedback intensities exceeding the very‐weak‐feedback regime.

To study the self‐mixing signal further, a gold‐coated high‐reflectivity external mirror was used to inject a larger amount of feedback into the THz QCL FC (**Figure** **3**a). In contrast to the experiments presented in Figures 1 and 2, a piezoelectric motor was used to vibrate the mirror by ≈1.5 µm and modulate the phase of the feedback (at ≈23 kHz), but leaving the amplitude unchanged. This led to lock‐in detection of a small (≈100 µV–0.1 mV) self‐mixing signal superimposed on the much larger (≈10 V) QCL contact bias. It should be noted, however, that, in this experimental configuration, the beatnote cannot be reliably recorded at each mirror step owing to the fast frequency shift induced by the change in the feedback phase.

Figure 3b shows the self‐mixing signal amplitude as a function of time, obtained by scanning the delay line along a 1.3 mm path with steps of 2.2 µm, and acquiring data with FC_2_ driven at three distinctive currents. In the right column, an expanded section is shown over a 20 ps range to illustrate the THz field oscillations in detail. The different envelopes of the recorded THz field reflect the multimode emission of FC_2_. The signal at 306 mA has 10x higher peak values than those obtained at 390 and 480 mA. Interestingly, higher intensity self‐mixing signals can also be observed in a number of very narrow current ranges, typically tens of µA wide, at the transition between different coherence states signaled by a consistent change in the beatnote linewidth. The latter, for example, nearly halves (see Figure S6, Supporting Information) passing from 300 to 306 mA, where we observe a >10 times increase in signal amplitude. On the contrary, conditions of broad beatnote (linewidth >1 MHz) correspond to noisy, almost undetectable self‐mixing signals (SNR<2).

The normalized amplitude spectra (Figure 3c) of these time‐domain traces reflects the modes of FC_2_ that are directly contributing to the self‐mixing signal. The principal modes are centered around 3 THz, as expected based on FTIR spectra in Figure 1c. We also observe, spectral replicas at higher harmonics in all three cases—these are related to the shape of the self‐mixing fringes and show a complex, nonsinusoidal dependence on the external cavity length, differing from simple sinusoids owing to higher levels of feedback.^[^ ^46^ ^]^ Furthermore, at low frequencies, modes in the tens and hundreds of GHz are visible in all cases. These originate from intracavity difference‐frequency generation.^[^ ^57^ ^]^

To demonstrate the capability of our FC‐based near‐field THz nanoscope to perform hyperspectral nanoimaging, we investigate the near‐field response of a 90 nm thick Bi_2_Se_3_ flake, which is a well‐known topological insulator^[^ ^58^ ^]^ with infrared active phonons in the 2–3 THz range. The target sample consisted of several flakes of Bi_2_Se_3_, deposited on a SiO_2_/Si (350 nm/300 µm) substrate with a set of lithographically defined 45 nm thick gold markers. The Raman spectra of the Bi_2_Se_3_ are shown in Figure S8 (Supporting Information).

To exploit its full spectral coverage, phase‐stability (given the 27 kHz beatnote linewidth in Figure S6, Supporting Information) and its maximum output power (≈6 mW, see Figure S5b, Supporting Information), FC_2_ was driven at 480 mA and coupled with the s‐SNOM tip (Figure 1a). The near‐field nature of the recorded self‐mixing signal is testified by the exponential decay of the retraction curves (Figures S9 and S10, Supporting Information) for high order *n* harmonics, *n* > 1. A spatial resolution of ≈175 nm (≈160 nm) was obtained with a 40 nm radius tip (10 nm radius tip), see Sections 9 and 10 of the Supporting Information, corresponding to a fraction <*λ*/570 (*λ*/625) of the THz wavelength *λ* = 100 µm, for the third order harmonics *V*_3_ = s_3_ exp(*i*Φ_3_), with amplitude s_3_ and phase Φ_3_, which is chosen as a good compromise between far‐field background suppression and SNR.

In the s‐SNOM experiment, the feedback is dominated by the far‐field backscattered light from the tip cantilever and diffraction‐limited focusing on the sample, controlling the mode coherence of the emitted modes. Therefore, the near‐field scattering signal measured while scanning the tip over the sample originates from the same impinging field, and mapping the sample in the near field is not expected to alter the QCL comb output after backscattering light in the cavity intracavity.

**Figure 4:**
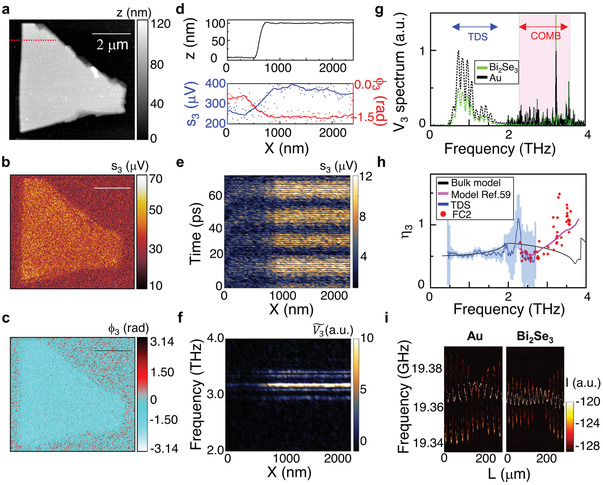
Hyperspectral THz near‐field imaging with a FC. a) Topography of a 90 nm thick Bi_2_Se_3_ flake, deposited on a SiO_2_/Si (350 nm/300 µm) substrate. b,c) Third‐order near‐field self‐mixing signals, acquired with FC_2_ driven at a 480 mA current and with a fixed delay stage position, *L* = 400 µm. b) Third‐order near‐field self‐mixing signal amplitude, s_3_, and c) phase,  Φ_3_. d) Line profiles of sample height z, s_3,_ and  Φ_3_ extracted by sampling a–c) along the red dotted line in a). e) Self‐mixing fringe map acquired as a function of position along the red dotted line in a), and scanning the optical path length *L* at each position, *X*. f) Power spectrum of *V*_3_ as a function of the position *X*, obtained by Fourier transforming the data in e). g) Near‐field *V*_3_ spectra of Bi_2_Se_3_ (green) and Au (black) measured by self‐mixing interferometry (solid lines) with a 200 ps scan and by THz‐TDS (dotted lines, details in Figures S15 and S16, Supporting Information), both normalized to the maximum measured on Au. The pink shaded area indicates the experimental spectral bandwidth of FC_2_ during the present experiments. h) Contrast *η*_3_ obtained as the ratio between the spectrum from Bi_2_Se_3_ and from Au measured by THz‐TDS (blue line and error bars evaluated as standard deviation on the averaged spectra where <0.5), self‐mixing interferometry (black dots), compared to the expected contrast for bulk infrared active phonons of Bi_2_Se_3_ (red line) and hyperbolic phonon polaritons from ref. [59] in flake of thickness 90 nm and 290 meV chemical potential (magenta line). i) Feedback‐induced variations of the intermode beatnote as a function of *L* during the measurements on Au (left panel) and Bi_2_Se_3_. (right panel).

**Figure** **4**a–c shows a) the topography of a 90 nm thick flake and the third harmonic b) amplitude s_3_ and c) phase Φ_3_ self‐mixing maps with a fixed delay line position. Line profiles obtained by cutting the maps along the red line sketched in Figure 4a are reported in Figure 4d. It is worth mentioning that the self‐mixing signal at a fixed delay results from the weighted average of a number of different modes of the QCL FC_2_ spectrum. In order to spectrally resolve the near‐field response of the flake, we acquired the self‐mixing fringes over a 70 ps range moving along the red line in Figure 4a. The resulting map (Figure 4e) reports the time traces of s_3_ as a function of the position X on a 2 µm long line. The Fourier transform over the time provides the near‐field THz spectrum at each sample position X (Figure 4f) including components of the emission frequencies of FC_2_ in the range 2.9–3.5 THz. The spectral phase of the Fourier transform of V_3_ can be found in Figure S13 (Supporting Information). The time domain and frequency domain maps show a visible signal increase at the transition from the substrate to the Bi_2_Se_3_ flake (X = 900 nm). The amplitude and phase of the different spectral components remains constant on the flake demonstrating its topographic and chemical homogeneity.

To retrieve quantitative information about the dielectric properties of Bi_2_Se_3_, we consider its spectral contrast with respect to Au, an ideal reference material owing to its almost flat reflectivity profile in the THz range.^[^ ^60^ ^]^ Figure 4g shows the self‐mixing spectra (*V*_3_) acquired on Au (black) and Bi_2_Se_3_ (green), extracted from the time traces acquired with our 6 mW emitting FC_2_, spanning over an extended 200 ps range to increase the spectral resolution (Figure S15, Supporting Information), and compared with similar traces measured with a commercial THz‐TDS system coupled to the s‐SNOM (see Figures S16 and S17, Supporting Information for details). Remarkably, the devised FC‐based nanoscope largely extends the frequency range covered by currently available THz‐TDS hyperspectral nanoimaging systems (0.4–1.8 THz) that also rely on very low (µW) optical power photoconductive antennas.

Clear differences between the hyperspectral near‐field response of Au and Bi_2_Se_3_ are seen. While in the range accessible with THz‐TDS the extrapolated near‐field contrast *η*_3_ is rather flat (see Figure 4h), a complex spectral dependence with a minimum at ≈2.5 THz, and *η*_3_ > 1 above 3.3 THz is revealed using FC_2_. The experimental contrast *η*_3_ is compared in Figure 4h with 3D bulk calculations of the near‐field contrast (black line), based on a well‐established finite dipole approximation (extensive details in ref. [36]), considering multiple longitudinal optical (LO) and TO phonon modes. The peaks in the near‐field scattering intensity of Bi_2_Se_3_ occur within the frequency band $\nu_{\mathrm{to}}^{⊥}$<*ω*<$\nu_{\mathrm{to}}^{//}$ identified for the long‐wavelength transverse optical (TO) phonon modes^59^: the E_u_ TO ( $\nu_{\mathrm{to}}^{⊥}$= 1.92 THz) and the A_2u_ TO ( $\nu_{\mathrm{to}}^{//}$= 4.05 THz) phonons, involving atomic vibrations in the plane orthogonal (⊥) and parallel (//) to the trigonal *c*‐axis, respectively, which clearly appear activated.

The bulk model (black curve, Figure 4h), which predicts a minimum at 3.7 THz, is not able to capture the *η*_3_ measured with our FC s‐SNOM, which instead, in the investigated 2.29‐3.60 THz range, is very well replicated by including^[^ ^59^ ^]^ deeply subdiffractional, highly directional hyperbolic phonon polaritons interacting with the electrons of the topological surface‐states of Bi_2_Se_3_ flakes, which can be activated by the s‐SNOM tip (magenta line in Figure 4h).^[^ ^61^, ^62^ ^]^

We also measured the intermode FC beatnote map as a function of *L* on both Au (Figure 4h) and the Bi_2_Se_3_ flake (Figure 4i), and observed an unexpected behavior as the feedback phase was varied. In both cases, the beatnote splits into two separated lines, which periodically merge into a more intense single beatnote. The amplitude of the frequency shift of each line is larger on Au than on Bi_2_Se_3_, confirming the different near‐field responses of the two materials, with the single beatnote condition more stable during the measurements on Bi_2_Se_3_. Comparing the self‐mixing signal amplitude to the beatnote parameters recovered from a Lorentzian fit of each beatnote spectrum, we find the same correspondence already observed at 328 mA (Figure 2), described in detail in Section 2, Supporting Information. The overall behavior can be associated with the interplay between the Lang–Kobayashi shift of the two main subcombs (Figure 1c), which share very similar mode spacing, leading to mode pulling of the respective beatnotes. By changing the feedback phase, the coherence state of the FC generating these beatnotes can then be controlled.

To demonstrate the reproducibility of our results, we also performed s‐SNOM imaging using FC_1_ to replace FC_2_ (Section 9, Supporting Information). We verified that operating over a broad current range with a single beatnote enhances the quality of the near‐field THz maps, and guarantees long‐term stability of the self‐mixing signal against small thermal and mechanical drifts.

Remarkably, our experiments prove that the phase coherence of the emitted modes, specific of the comb, is the essential requisite for self‐mixing interferometry detection with our multimode THz‐QCLs, disclosing exceptional application for hyperspectral near‐field imaging at THz frequencies and for control of comb operation of THz‐QCLs. Indeed, a simple multimode QCL operating in self‐detection mode cannot provide the SNR required for any near‐field microscopy, spectroscopy, or sensing applications (see Figure S18, Supporting Information).

## 3 Discussion

Our results demonstrate the ability to perform phase‐sensitive hyperspectral THz nanoscopy, while simultaneously providing a powerful tool to study the complex coherence dynamics of THz FCs, enabling identification of stable regimes for phase‐locking FC modes. Individually, these aspects have been investigated previously through THz TDS s‐SNOM imaging^[^ ^37^, ^38^, ^63^ ^]^ and SMIB experiments,^[^ ^26^ ^]^ although the study of EOF^[^ ^26^, ^28^, ^29^ ^]^ on multimode THz QCLs has been limited to the induced frequency shift of intermodal beatnotes. Hyperspectral imaging of the fifth harmonics signal is here achieved with a 7 ms integration time per fringes point (see Figure S13, Supporting Information), that allows the acquisition of the entire near‐field spectrum, with the same resolution as Figure 4g, in less than 5 s per pixel, while for state‐of‐the‐art TDS‐SNOM only up to the second harmonics signal has been reported as obtained with 13.5 s per pixel and same 40 nm‐radius AFM tip.^[^ ^63^ ^]^

In general, the effect of EOF on the coherence of a comb has largely been neglected under the assumption that either EOF results in a collective variation of the intermodal spacing or a destruction of the FC regime. In this work, we have shown that this is not the case. Indeed, the coherence of the comb can actually be enhanced when the phase and the intensity of the feedback are set appropriately.

These results are extremely relevant to the self‐mixing response of a THz QCL FC, which represents the very core of our imaging technique, and is significantly affected by the coherence of its spectrum. Specifically, the high SNR attainable (>20 for *V*_2_ with 100 ms integration time), when the QCL exhibits a single beatnote, can be attributed to the extension of the fixed‐phase relation between the modes of a comb to the self‐mixing signals generated by each mode. In fact, each self‐mixing contribution can sum constructively as long as the modal phase difference is well defined. Such a condition can only occur if the QCL is in a stable FC regime. In contrast, incoherent contributions would lead to a weaker self‐mixing signal with small amplitude dependence on the feedback phase.

We have clearly demonstrated that hyperspectral s‐SNOM imaging can be successfully implemented by exploiting the mode coherence of a quantum‐engineered laser source with a tunable‐by‐design spectral bandwidth. This highlights that QCL FCs are highly advantageous, compared to more generic multimode QCLs, for any self‐mixing interferometry application, in both far‐field and near‐field configurations. We also note that our detection scheme provides a NEP ≈400 pW √Hz^−1^ at frequencies >2.7 THz, which have previously been unexplored in THz‐QCL‐based self‐mixing (see Section 14, Supporting Information).

As a further consideration, previous SMIB experiments have shown that the frequency shift of the beatnote can be described by the average Lang–Kobayashi shift of all the comb mode pairs.^[^ ^26^ ^]^ This means that for combs comprising many modes, which is the preferred condition for metrological spectroscopic and imaging applications, the beatnote shift becomes less sensitive to the feedback phase as individual contributions are more likely to cancel each other out. The same information loss does not occur with the beatnote intensity variation that we have demonstrated. In this case, no averaging is involved and all contributions sum. Moreover, we have shown that the amplitude distribution of the beatnote shift spectrum significantly differs from that of the other observables. Whether this is due to the structure of the gain region or to some more fundamental nonlinear intracavity phenomena (FWM and related 3rd order nonlinear susceptibility), we argue that the variation of the beatnote intensity is a more suitable observable for the study of THz FCs than SMIB experiments.

## 4 Conclusions

We have reported a coherent hyperspectral nanoimaging system, exploiting the multimode coherent emission of THz QCL FCs. This has enabled mapping of a thin flake of Bi_2_Se_3_ over a 1.5 THz spectral range with submicrometer spatial resolution. Further increase of the SNR can be achieved by operating the tip at larger tapping amplitudes and by choosing AFM tip of larger radius, the latter at the expense of a smaller spatial resolution.^[^ ^64^ ^]^ We have also demonstrated the complex sensitivity of the THz‐QCL FC coherence to optical feedback, which can be used to control the amplitude, linewidth, and central frequency of the FC intermode beatnotes, and enable stable coherence between modes to be achieved, where it spontaneously does not happen. The technique opens up the possibility of dual‐comb detection schemes,^[^ ^12^, ^13^, ^14^, ^65^, ^66^ ^]^ where a second THz QCL, with slightly different repetition rate, is used to down‐convert each individual THz line of the QCL under EOF into the radiofrequency range. This would allow retrieval of the full amplitude and phase information of each mode almost instantaneously. Remarkably, we demonstrate a new type of characterization of QCL FCs using s‐SNOM as a tool. We then show that the combined system also works in reverse, i.e. as a near‐field microscope and we demonstrate hyperspectral near‐field nanoscopy in a frequency range (>2 THz), traditionally chartered only by bulky free‐electron lasers,^[^ ^39^ ^]^ envisioning a broad application potential in a plethora of interdisciplinary fields as biophysics, for chemical selective nanoimaging of biomolecules (e.g., nucleobases^[^ ^67^ ^]^ and single cells^[^ ^68^ ^]^) or viruses (e.g., targeting the high‐order structure of capsid proteins as those of coronavirus),^[^ ^69^ ^]^ cultural heritage for capturing resonant pigments^[^ ^70^ ^]^ for nondestructive study of artworks, process, and quality control of nanoplastics for polymer identification of interest for environment conservation.^[^ ^71^ ^]^ The concept is also scalable to the mid‐IR,^[^ ^20^ ^]^ to larger THz frequencies (6–7 THz) in a difference frequency generation architecture and at room temperature,^[^ ^72^, ^73^ ^]^ or in a compact Peltier cooler configuration, the latter promising a fast translation in a commercial system for real‐time in situ applications.^[^ ^74^ ^]^

## 5 Experimental Section

### Fabrication of the THz QCL‐Based Frequency Combs

The GaAs/AlGaAs heterostructure of FC_1_ and FC_2_ comprises three active regions, exploiting alternating photon‐ and longitudinal optical (LO) phonon‐assisted interminiband transitions, individually designed to operate at different central frequencies (2.5, 3.0, 3.5 THz). The two ARs differ in the doping concentration (*n*_d_ = 3.2 × 10^16^ cm^−3^ for FC_1_, and *n*_d_ = 4.0 × 10^16^ cm^−3^ for FC_2_), which is optimized to achieve a flat gain bandwidth (and uniform power output across the whole spectrum), and to obtain two high dynamic current density ranges (*J*_max_/*J_t_*_h_ = 2.9). Both lasers were fabricated in a metal–metal waveguide configuration with a set of nickel side absorbers^[^ ^75^ ^]^ that have the specific purpose of inhibiting lasing from higher order lateral modes.

To fabricate the lasers, the bottom highly‐doped GaAs contact layer was first exposed through a combination of initial mechanical thinning and subsequent selective wet‐etching, after removal of the Al_0.5_Ga_0.5_As etch‐stop layer. The 17 µm thick active region was then defined by dry etching using inductively‐coupled plasma reactive ion etching (ICP‐RIE). Dry etching allows almost vertical sidewalls, which result in uniform current injection and reduced lateral optical scattering. A Cr(10 nm)/Au(150 nm) top contact was next defined by a combination of optical lithography (SUSS MicroTec MJB4), thermal evaporation and lift‐off. The top contact was intentionally patterned narrower than the ridge top surface, allowing thin nickel side‐absorbers (setbacks) to be deposited via optical lithography on the uncovered portion of the ridge surface, using a laser writer (MicroWriter ML3 Durham Magneto Optics).^[^ ^75^ ^]^ The side‐absorbers are 5 µm wide and 5 nm thick for FC_1_, and 2 µm wide and 5 nm thick for FC_2_. Laser bars were finally cleaved, mounted on a copper bar with an indium‐based thermally conductive adhesive paste, wire bonded with an ultrasound wedge bonding and connected to high frequency coplanar waveguides. FC_1_ is 50 µm wide and 3.8 mm long, FC_2_ is 50 µm wide and 2.3 mm long.

### SMIB Experimental Setup

The THz QCL was operated in CW, with radiation collimated by a 50 mm focal length parabolic mirror, and focused by two parabolic mirrors (f/1 = 2 in.) on an AFM tip (25PtIr300BH‐40, 80 µm long Rocky Mountain Nanotechnology, 40 nm apex‐radius) of a s‐SNOM microscope from Neaspec/Attocube. The THz radiation was concentrated at the tip apex by lightning‐rod effect and adiabatic compression. The radiation backscattered by the tip was then coupled into the laser cavity and the self‐mixing signal detected as a perturbation (Δ*V*) to the QCL contact voltage. The AFM tip was operated in tapping mode at a frequency Ω ≈22 kHz, such that the near‐field contribution to the self‐mixing signal could be isolated by lock‐in detection (with a UHFLI from Zurich Instruments) at the tapping frequency harmonics nΩ, with *n* = 2,..,5. Simultaneously, a wire‐based optical antenna in copper, placed in proximity of the cryostat (≈3–4 cm away from the QCL), was used to collect the intermode beatnote signal isotropically radiated into free space by the THz QCL FC, detected using a spectrum analyzer (Rohde and Schwarz FSW43). The beatnote signal was also alternatively monitored through a bias‐T directly connected to the QCL output. An optical delay‐line, equipped with a translation stage with 0.1 µm resolution (IntelliStage M521‐Physik Instrumente), was used to vary the optical path and control the phase of the optical feedback.

## Conflict of Interest

The authors declare no conflict of interest.

## Author Contributions

V.P. and E.A.A.P. contributed equally to this work. M.S.V. conceived the concept. V.P. and E.A.A.P. set up the experiment, acquired and interpreted the experimental data and characterized the QCL frequency combs. L.V. fabricated the QCL frequency combs; L.L., A.G.D., and E.H.L. grew the QCL frequency combs. The manuscript was written and the data interpreted by V.P., E.A.A.P., and M.S.V. All authors contributed to the discussion and to the final manuscript. All work was coordinated and supervised by M.S.V.

## Supporting information

Supporting Information

## Data Availability

The data that support the findings of this study are available from the corresponding author upon reasonable request.
