# Supplementary material for: Self‐Induced Phase Locking of Terahertz Frequency Combs in a Phase‐Sensitive Hyperspectral Near‐Field Nanoscope
Source: Adv Sci (Weinh). 2022 Jun 16;9(28):2200410. doi: 10.1002/advs.202200410 (PMC9534969; doi:10.1002/advs.202200410)
Supplement: Supplementary file 1 — Supporting Information [file ADVS-9-2200410-s001.pdf]

ADVANCED SCIENCE
Open Access

Supporting Information

for *Adv. Sci.*, DOI 10.1002/advs.202200410

# Self-Induced Phase Locking of Terahertz Frequency Combs in a Phase-Sensitive Hyperspectral Near-Field Nanoscope

*Valentino Pistore, Eva Arianna Aurelia Pogna, Leonardo Viti, Lianhe Li, A. Giles Davies, Edmund H. Linfield and Miriam Serena Vitiello**

**Supplementary information**

# Self-induced phase locking of terahertz frequency combs in a phase-sensitive hyperspectral near-field nanoscope

**Valentino Pistore,[1*] Eva A. A. Pogna,[1*] Leonardo Viti,[1] Lianhe Li,[2] A. Giles Davies,[2] Edmund H. Linfield[2] and Miriam S. Vitiello [1]**

*[1]NEST, CNR-Istituto Nanoscienze and Scuola Normale Superiore, 56127 Pisa, Italy*
*[2]School of Electronic and Electrical Engineering, University of Leeds, Leeds LS2 9JT, UK*

** these authors, listed in alphabetic order, contributed equally to the work*

## 1. Effect of the optical feedback on the beatnote map

The beatnote maps of $FC_1$ and $FC_2$ are presented in Fig. S1 when the devices are coupled to the AFM tip. Weak external (amplitude/phase) optical feedback can affect the beatnote frequency, intensity and linewidth, compared to the case with no feedback (Figs 1d-e, main text). These changes can be seen more clearly for $FC_1$ owing to the limited frequency shift (~0.4 GHz) of the beatnote when the drive current is changed (Fig. S1a). In contrast, the beatnote of $FC_2$ appears over a broad frequency range (>2 GHz), which limits the visibility of the feedback-induced frequency shift. Nevertheless, changes in the beatnote intensity can be identified in both maps. The linewidth variation induced by the feedback cannot, however, be seen due to the 'binning' of the beatnote spectra which is required to display the maps.

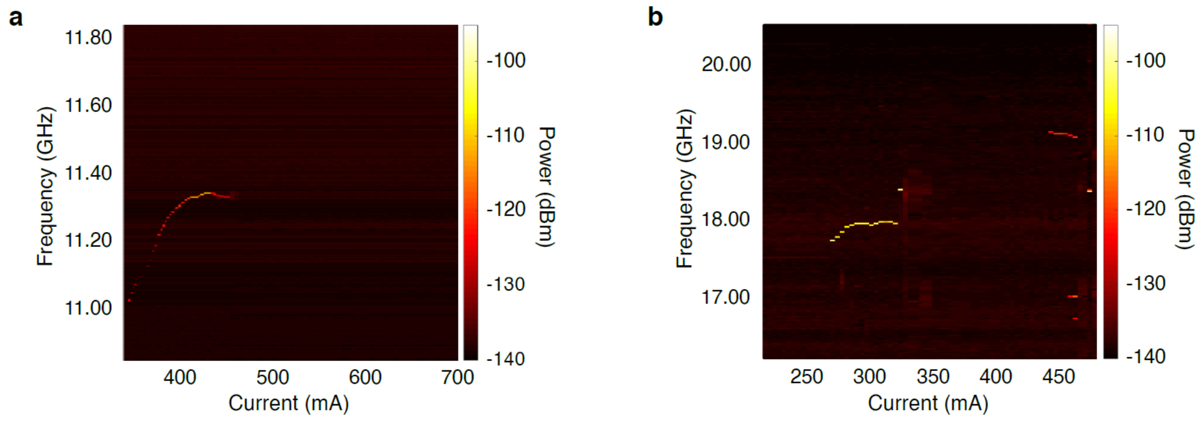


**Figure S1**: **Beatnote maps with external optical feedback. a**, Beatnote map of $FC_1$ recorded as a function of the bias current with the QCL experiencing external optical feedback from the AFM tip. **b**, Beatnote map of $FC_2$ recorded as a function of the bias current with the QCL experiencing external optical feedback from the AFM tip.

## 2. Effect of the optical feedback on the intermodal beatnote

The beatnote spectrum of the THz QCL frequency comb $FC_2$, coupled to the AFM tip of the s-SNOM system, was recorded at each step of the delay line scan (Fig. S2a). The 8 cm optical path length variation corresponds to a spectral resolution of 3.75 GHz for the retrieved spectra (Fig. 2b, main text). Individual beatnotes, recorded during the scan, are fitted with Lorentzian functions to extract the beatnote area, the full-width-at-half-maximum (FWHM) and central frequency. These observables provide information on the beatnote power (area), FC coherence (FWHM) and intermodal spacing (central frequency). In order to understand between the variation of these quantities, and the self-mixing terminal voltage signal, with feedback phase, we consider each parameter in the time domain.

The self-mixing voltage signal in Fig. S2b, referenced to the tapping-mode frequency (22.2 kHz), and filtered above 7.5 THz to remove high-frequency noise, displays a set of fringes of dissimilar (and not purely sinusoid) shape. This is a consequence of the degree of feedback sensed by the QCL. In fact, as the spectrum comprises many modes, the shape of the fringes will also depend on the contribution of each of the FC frequencies and their relative phase relation, different from the case when self-mixing is measured with pure, single-mode QCLs[1,2].

Figs S2c-d show the FWHM and the intermode beatnote power. The pink dashed lines in the figures are used as a visual support to identify specific points corresponding to maxima or minima of the curves. The FWHM curve (Fig. S2c) appears to be in phase with the self-mixing signal, whereas the beatnote power (Fig. S2d) is in counter-phase. This means that the self-mixing signal is maximized when the beatnote is broadened and its intensity is reduced. The interaction of the intracavity field with the external feedback, however, does not destroy the comb coherence –the oscillatory behaviour of both the FWHM and the beatnote power shows that changing the feedback phase can reverse the phenomenon. Moreover, one can control the coherence properties of the comb by exploiting the optical feedback.

As a further remark, it is important to note that both the FWHM and the beatnote power only depend on the phase-locked modes that generate the recorded beatnote. The self-mixing signal, on the other hand, is generated by all modes in the spectrum of the heterogeneous QCL. According to our previous observations, however, the self-mixing fringes are highly correlated to the the beatnote. This is, in part, expected since the presence of a single, narrow beatnote is a strong indication of a stable FC regime in a THz QCL. Without a fixed phase relation between the modes, the self-mixing signal would result in an

aperiodic waveform. Indeed, in the complete absence of coherence, ie when no beatnote is generated by the QCL, the self-mixing response of the device to feedback may result in a chaotic behaviour, characterized by no distinguishable variation with external path length and a significant overall decrease in SNR. This behaviour is clearly visible for $FC_2$ in the current range (330-440 mA) where $FC_2$ does not exhibit a beatnote (Fig. 1e); the self-mixing signal then is completely dominated by noise.

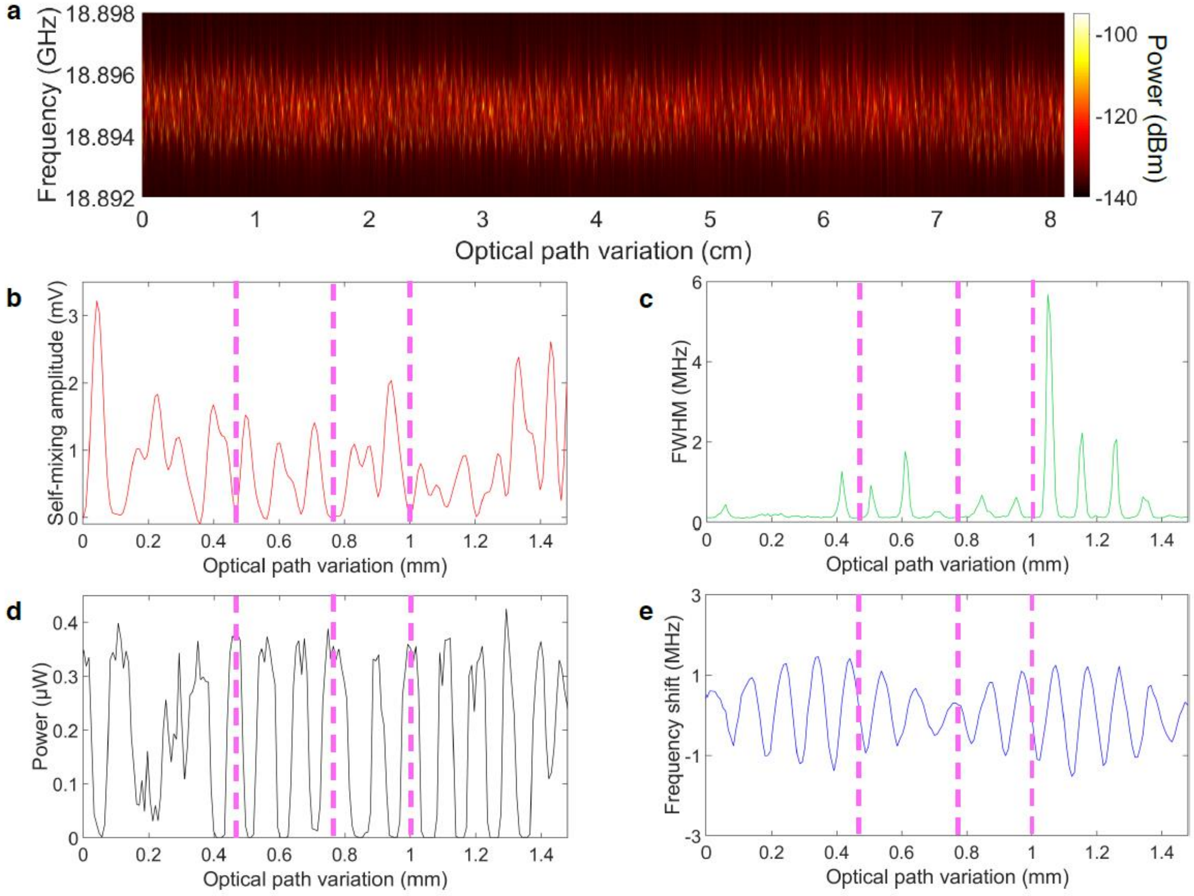


**Figure S2**: **Time-domain comparison of the self-mixing signal with beatnote parameters. a,** Beatnote spectrum map of $FC_2$ recorded as a function of the optical path variation with steps of 8 μm. Fig. 2c in the main text corresponds to a 1.5 mm long part of this map. **b,** Self-mixing signal corresponding to the time range of Fig. 2c, main text. **c-d-e,** (c) FWHM, (d) power and (e) central frequency variations obtained from fitting the beatnote spectra of Fig. 2c. The pink dashed lines provide a visual aid to identify a number of local maxima and minima in the time traces.

The central frequency shift trace (Fig. S2e) appears to follow the same behaviour as the other observables when observed for short time ranges of a few ps. However, this is not the case for the whole trace, as a dephasing is observed on longer timescales. This is consistent with our observation that the modes determining the frequency shift are not necessarily those contributing most significantly to the self-mixing signal.

## 3. Spectral comparison of the SMIB and FTIR spectroscopies

The spectra retrieved by Fourier transforming the beatnote area, power and central frequency (Fig. 2c, main text) display modes whose intensity is not directly proportional to the intensity of the emitted FC modes. Similar to the self-mixing terminal voltage, it is expected that the retrieved mode intensity is proportional to the ratio of the feedback intensity and the intracavity intensity at the mode frequency, when the device operates in the linear regime. As a result, the amplitude distribution in the spectrum can be considerably more homogeneous than that retrieved from an FTIR spectrum, if the material providing the feedback has a flat reflectivity profile in the emission range of the laser.

The FTIR spectrum of $FC_2$, corresponding to the retrieved spectrum shown in Fig. 2c, is shown in Fig. S3a. Fig. S3b then shows the comparison between the beatnote power and FTIR spectra. A linear scale is chosen specifically to identify the main modes contributing to the change of the beatnote during the delay line scan. The main modes above 3 THz in the FTIR spectrum (red trace, Fig. S3b) are also visible in the power spectrum (blue trace), whereas other minor modes also appear with increased intensity, as previously explained. It is worth noting that the mode located at 2.73 THz is the most intense below 3 THz for both spectra. This suggests that the power variation can be used to identify correctly the modes that principally contribute to the generation of intermode beatnotes. In contrast, the Fourier transform of the central frequency shift (Fig. S3c, blue trace) clearly overestimates the mode at 2.84 THz. As such, the modes contributing to the shift of the beatnote central frequency are not necessarily those having the highest intensity in the emission spectrum. This observation is in contrast with previously reported results[3,4].

The self-mixing terminal voltage spectrum (Fig. S3d, blue trace) displays a significantly lower SNR than is obtained by fitting the beatnote. This is partly due to the contribution of the incoherent parts of the emission spectrum, as argued in Section 2. Regardless of the higher noise floor, a visible similarity with the power variation spectrum can be seen. In fact, the far-field contribution to the scattered radiation is still visible at the employed tip tapping frequency (22.2 kHz). In order to remove the far-field contribution, one must consider higher harmonics of the tapping frequency. However, since the beatnote spectrum acquisition is not a referenced measurement, a meaningful comparison between the near-field components of the self-mixing and the beatnote parameters would require a more

sophisticated setup to access the very small near-field information encoded in the beatnote spectrum.

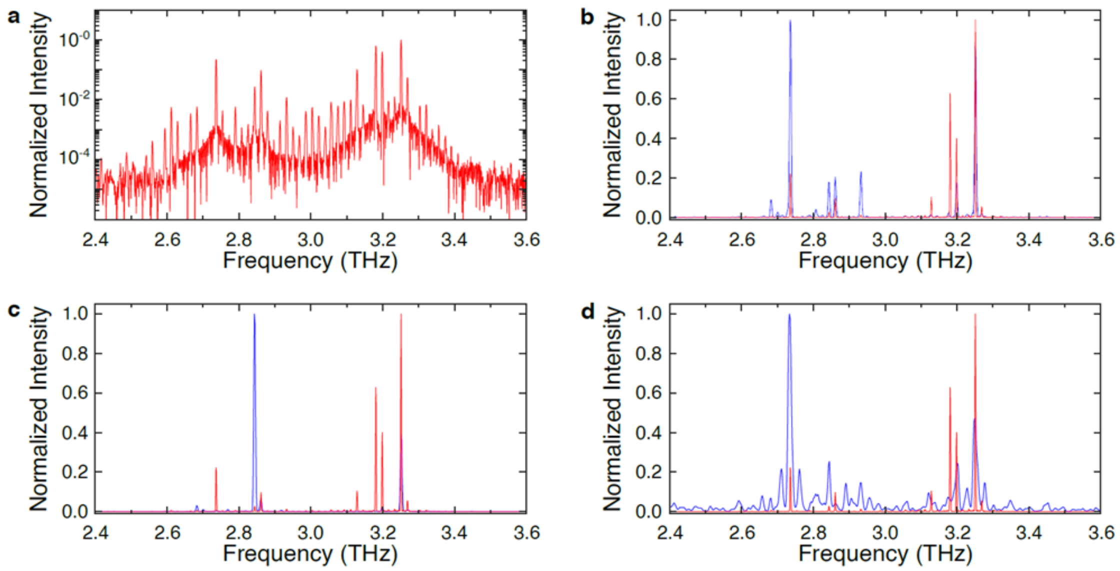


**Figure S3**: Comparison of FC2 FTIR spectrum with the spectra of the beatnote parameters. **a**, FTIR spectrum of $FC_2$ biased at 328 mA. In the following panels the same spectrum is presented on a linear scale. **b,** Comparison between the power variation of the beatnote (blue) and the FTIR spectrum (red). **c,** Comparison between the frequency shift spectrum (blue) and the FTIR spectrum (red). **d**, Comparison between the self-mixing terminal voltage spectrum (blue) and the FTIR spectrum (red).

In Figure S4, we provide the FTIR and BT shift spectra in logarithmic scale, showing that, despite the naturally higher noise floor of the BT shift spectrum, the bandwidth of the two spectra is the same.

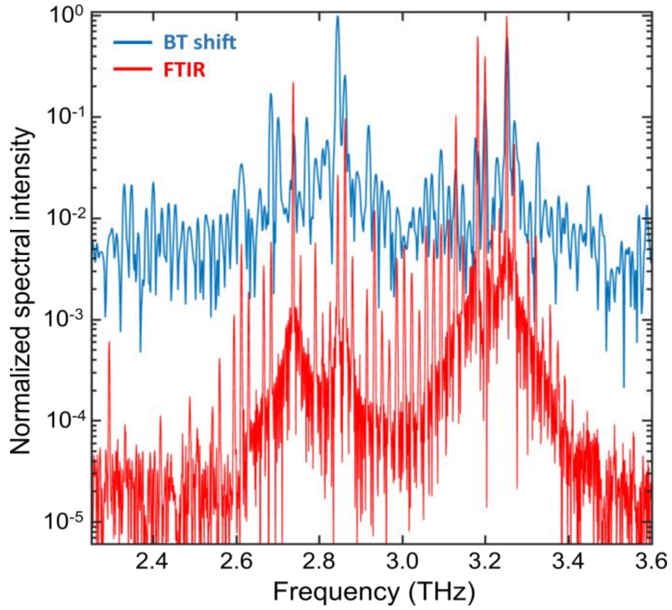


**Figure S4:** Spectra from Figure 2d in logarithmic scale showing that self-mixing signal and the beatnote shift have the same spectral bandwidth

## 4. Electrical characterization of the QCL FCs

Figures S5a-b show the continuous-wave voltage-current (blue traces) and light-current (red traces) characteristics of $FC_1$ and $FC_2$, respectively, measured at 18K. $FC_1$ is 3.8 mm long and 50 µm wide, $FC_2$ is 2.3 mm long and 50 µm wide. The threshold current densities ($J_{th}$ = 157Acm$^{-2}$ and $J_{th}$ = 170 Acm$^{-2}$) are almost comparable.

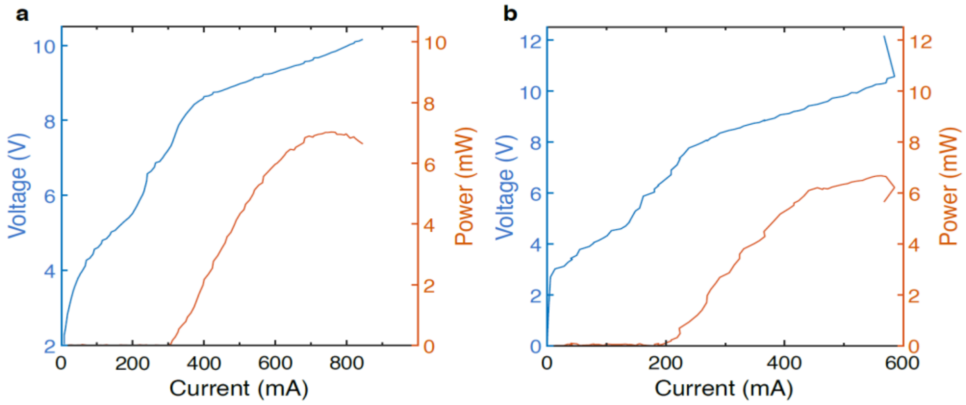


**Figure S5: Voltage-current density and light-current density characteristics. a**, Continuous wave current-voltage-power characteristics of $FC_1$. **b,** Continuous wave current-voltage-power characteristics of $FC_2$.

## 5. Intermode beatnote linewidth map of $FC_2$

Figure S6 shows the intermode beatnote linewidth (LW) map of $FC_2$: in the regime of stable comb operation, the beatnote LW ranges between 27 kHz and 90 kHz. We selected the two operational conditions identified by the shaded green area to perform our experiments. The high current regime (I = 480 mA) corresponds to a stable comb regime (single and narrow beatnote, LW = 27 kHz) with an emission spectra covering > 1THz bandwidth (Fig. 1c)

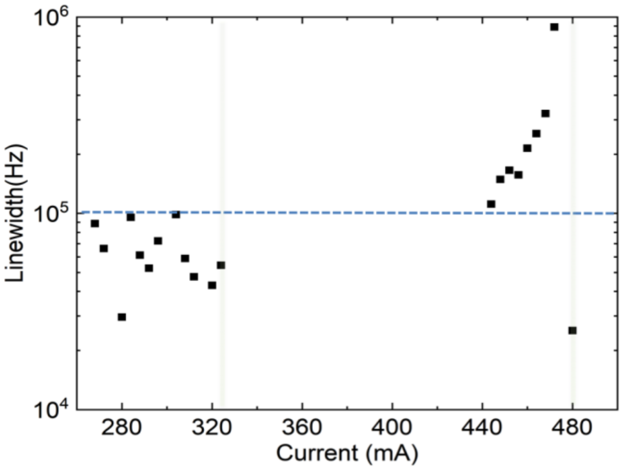


**Figure S6: $FC_2$ beatnote linewidth.** Intermode beatnote line width of $FC_2$ plotted as a function of driving current. The vertical green shaded areas mark the main current regimes investigated.

## 6. Comb coherence control by weak external optical feedback phase tuning

The coherence state of the spectrum of $FC_1$ can be controlled by tuning the phase of the weak optical feedback from the AFM tip. Figure S7a displays the beatnote spectra of $FC_1$, biased at 400 mA and at a heat sink temperature of 17.5 K, kept constant within 0.1K, to avoid variation in the emitted power, mode coherence and thermal expansion that may affect the cavity length and thus the feedback relative phase, when the feedback is blocked (blue curve) and when the feedback is enabled (red curve). Without feedback, the device exhibits two broad ($FWHM_1$ ~559 kHz, $FWHM_2$ ~1.79 MHz) beatnotes separated by less than 2 MHz, signature of the poor phase coherence among the THz modes of the QCL in such a specific bias point. As the feedback is enabled, the beatnote spectrum shows a single narrow (FWHM ~24 kHz) beatnote (red curve).

In fig. S7b, the beatnote spectra of $FC_1$, in the same experimental conditions as before, are reported as a function of the optical path variation due to the delay line movement. It is possible to observe two main regimes: between 0 and 40 µm, no beatnote is observed and the laser does not show a comb behaviour (panel c, green curve); between 40 and 120 µm a single narrow beatnote is present and the QCL then shows a stable comb behaviour (panel c, red curve).

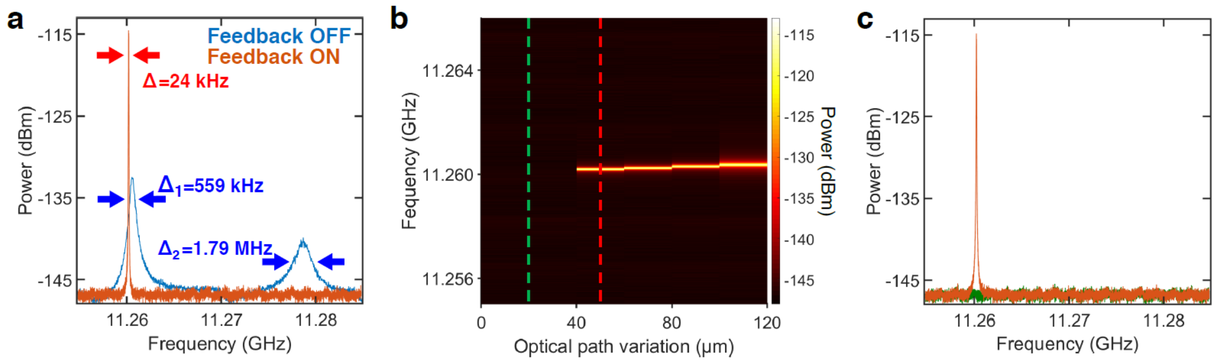


**Figure S7: Comb coherence control in $FC_1$**: **a**, beatnote spectra of $FC_1$ with (red curve) and without (blue curve) the feedback from the AFM tip, acquired at 400 mA and 17.5 K. The beatnote linewidth (Δ) is marked on the graph; **b**, beatnote spectra of $FC_1$ acquired as a function of the optical path variation; **c**, beatnote spectra of $FC_1$ for two different feedback phase delays, corresponding to the dashed lines of panel (b) of the same colours. The red curves in panels (a) and (c) represent the same beatnote spectrum.

Therefore, thanks to our technique one can easily manipulate the comb state of the laser either significantly enhancing the modes coherence or destroying it entirely, just by tuning the

feedback phase. Moreover, when at least some coherence between the modes already exists without the feedback, as in the case reported here, one can induce the formation of a spectral comb with an appropriate choice of the feedback phase. The proposed technique is therefore capable to extend the bias range in which the THz QCLs operate as combs, even in specific points in which dispersion compensation is inefficient.

### 7. Raman measurements

Figure S8 displays the micro-Raman analysis of the 90 nm thick $Bi_2Se_3$ flake mapped in the near-field (Fig. 4a). Fig. S8a is measured by exciting the flake at a 532 nm wavelength with an optical power of 100 μW and with a lateral spatial resolution of ~ 2 μm. The Raman spectrum reveals the presence of three Raman active modes which unequivocally[5] identify the material as $Bi_2Se_3$. These are out-of-plane $A_{1g}{}^1$, $A_{1g}{}^2$ and in-plane $E_g{}^2$ modes, located at 68.5 $cm^{-1}$, 128 $cm^{-1}$ and 172 $cm^{-1}$ respectively. The weak $E_g{}^1$ mode is absent, confirming the excellent crystalline quality of the investigated material.

The homogeneity of the response is evaluated from the map of Fig. S8b in which the red colour represents the intensity of the 68 $cm^{-1}$ mode and blue the intensity of the Si mode at 520$cm^{-1}$. The flake area can be easily identified from the surrounding Si substrate. As a comparison, an optical image is shown in Fig. S8c, which corresponds very well with shows with the recorded 68 $cm^{-1}$ mode in the flake area.

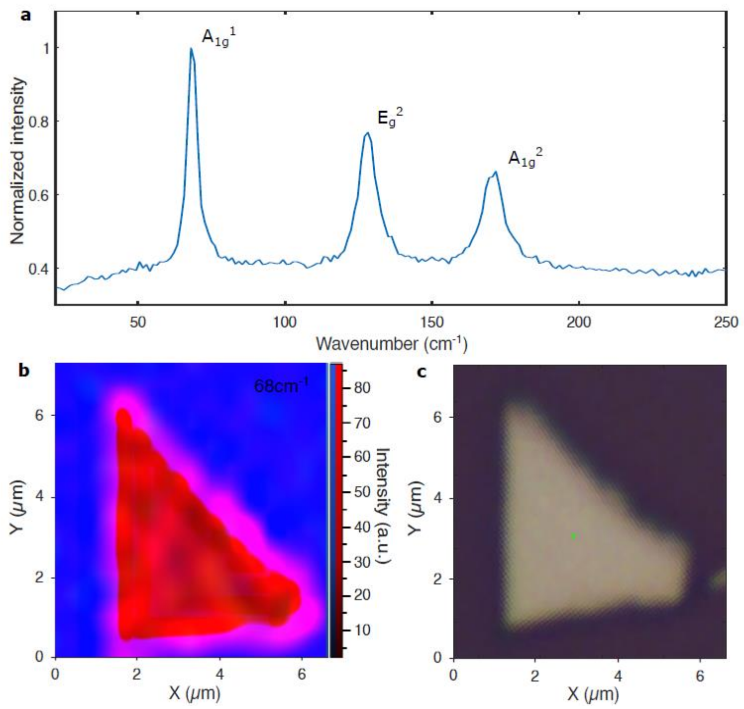


**Figure S8: MicroRaman characterization of the $Bi_2Se_3$ flake. a**, Micro-Raman spectrum of the 90-nm-thick $Bi_2Se_3$ flake. **b**, Micro-Raman point map displaying the intensity of the 68 cm$^{-1}$ peak in red and the 540 cm$^{-1}$ peak corresponding to the Si substrate in blue. **c**, Optical image showing the correspondence between the Raman map in (b) and the flake.

## 8. Retraction curve

In the THz nanoscopy experiment, the tip is operated in tapping mode with an oscillation frequency of 22.2 kHz. The near-field signal depends non-linearly on the tip-sample distance as is shown by the retraction curve reported in Fig. S9, measured on an Au marker with $FC_2$ driven at 300 mA. The near-field interactions decrease over distances comparable to the tip radius (40 nm), whereas the background varies on the radiation wavelength scale (tens of microns for THz frequency radiation). To remove the contribution of far-field scattering to the signal, harmonics of increasing order $n$ can be chosen. From Fig. S9a, we observe that the signal reaches the noise level at the limit of large sample-tip distances for n=3, testifying for an efficient far-field background removal. To find a good compromise between signal intensity and suppression of the background due to non-local scatterers, we select the third order harmonic n = 3 to analyse the near-field maps.

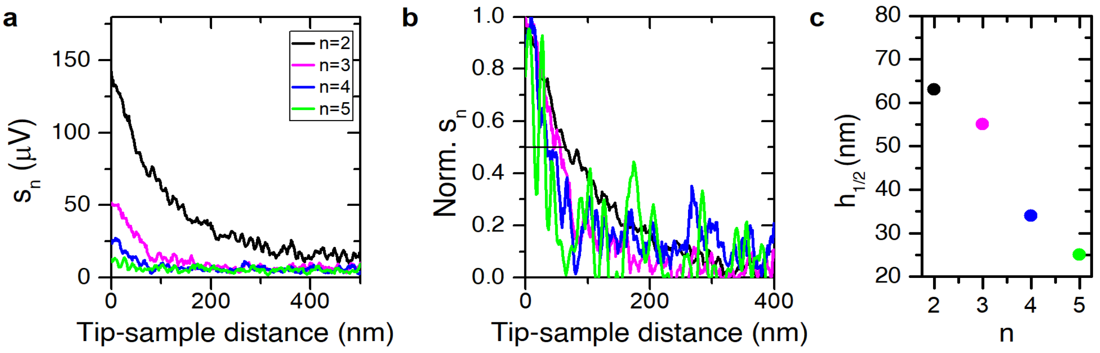


**Figure S9: Retraction curves measured on an Au marker with $FC_2$. a,** Retraction curves acquired when demodulating at the $n^{th}$ harmonic of the tapping frequency with n > 2, and with $FC_2$ driven at I = 328mA. The rapid signal increase as the tip approaches the sample testifies to the near-field nature of the detected self-mixing signal. On increasing the tip-sample distance, the signal reaches the noise floor at 40 nm for n=3, indicating that the contribution from far-field background radiation is negligible for n ≥ 3. **b,** Retraction curves from (a), normalised to the maximum. **c,** Tip-sample distance, $h_{1/2}$, at which the near-field signal halves as a function of the demodulation order, n, providing an estimate of the near-field vertical confinement.

From the distance, $h_{1/2}$, at which the normalized curves in Fig. S9(b) halve, we can quantify the near-field vertical confinement, and find $h_{1/2} \sim 50$nm for n=3.

Analogous retraction curves were acquired for $FC_1$, driven at 400 mA and approaching the s-SNOM tip to a 45 nm thick Au marker (Fig. S10). The tip used for the near-field experiment with $FC_1$ (25PtIr200BH-10 from Rocky Mountain Nanotechnology) has a smaller apex radius of 10 nm. In this case, we observe an efficient background removal already for n ≥ 2.

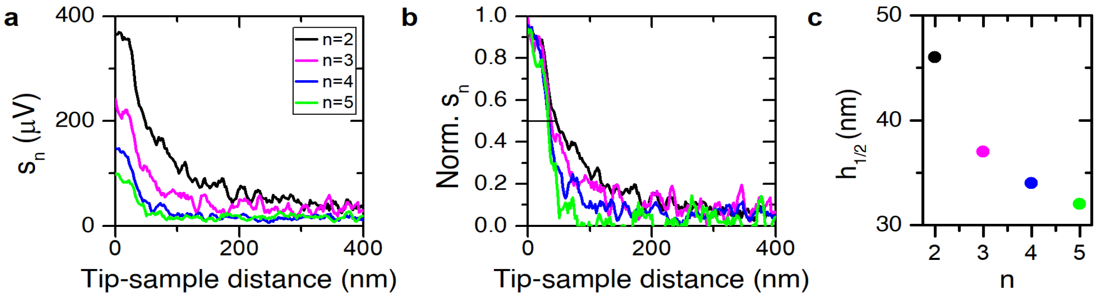


**Figure S10: Retraction curves measured on an Au marker with $FC_1$. a,** Retraction curves acquired when demodulating at the $n^{th}$ harmonics of the tapping frequency with n > 2, and with $FC_1$ driven at I = 400mA. The rapid signal increase as the tip approaches the sample testifies to the near-field nature of the detected self-mixing signal. On increasing the tip-sample distance, the signal reaches the noise level at 20 nm for n=2, indicating that the contribution from the far-field background radiation is negligible for n ≥ 2. **b,** Retraction curves from (a) normalised to the maximum. **c,** Tip-sample distance, $h_{1/2}$, at which the near-field signal halves as a function of the demodulation order, n, providing an estimation of the near-field vertical confinement.

## 9. Near-field imaging with $FC_1$

To validate the reproducibility of our nano-imaging technique with $FC_1$, we used the setup of Fig. 1a, with $FC_1$ driven in continuous-wave at 400 mA.

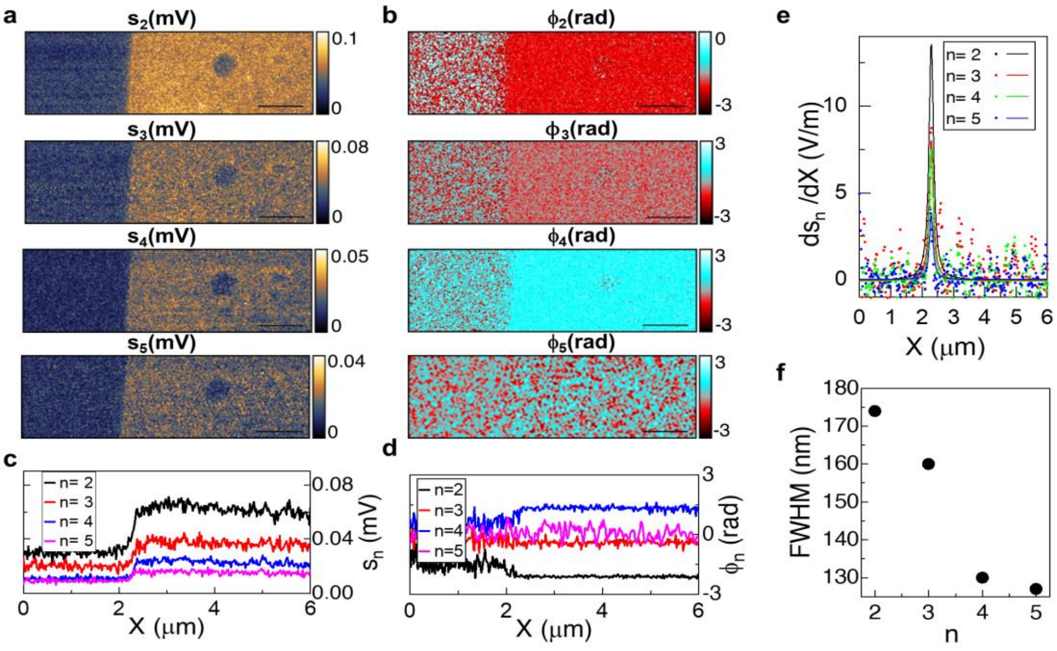

**Figure S11: Near-field imaging with THz-QCL $FC_1$. a-b,** Near-field self-mixing signals acquired with $FC_1$ driven at 400 mA for different demodulation orders (n=2, 3, 4, 5), separated into (a) amplitude, $s_n$, and (b) phase, $\phi_n$, components, obtained by scanning across a 45nm-thick Au marker (brighter region) on an undoped Si substrate. **c-d,** Line-profiles extracted as cross-sectional averages of the maps in (a)-(b) for (c) the signal amplitude, $s_n$, and (d) the phase, $\phi_n$. **e,** Line-spread function (LSF) obtained by evaluating the first space-derivative of the line profiles of signal amplitude in (c) (colored dots) together with the Gaussian fit (solid colored line) for demodulation orders n=2, 3, 4, 5. **f,** FWHM of the LSF of (e), extracted from the Gaussian fit, which can be assumed to be an estimate of the spatial resolution of our near-field imaging experiment

Under this condition, $FC_1$ operates as a stable FC showing a single and narrow[6] (10 kHz) beatnote (Fig.1c); the evolution of the intermode beatnote linewidth of $FC_1$ can be found in ref. 6. The PtIr-coated tip employed in this experiment had a nominal radius of 10 nm, a resonance frequency of 80 kHz, and operated with tapping amplitude of 540 nm.

We first characterized the signal amplitude $s_n$ (Fig. S11a), phase $\phi_n$ (Fig. S11b) and lateral resolution, $\Delta x$, by scanning across an interface between a 45 nm thick Au marker and the Si substrate, and keeping the external cavity length fixed. The line profiles (Figs. S11c-d), are extracted by averaging the maps of Figs. S11a and S11b along the vertical direction.

The spatial resolution was extracted as the FWHM of the line spread function (LSF), calculated by taking the derivative (Fig. S11e) of the line profiles of signal amplitude (Fig.

S11c) at different demodulation orders. The FWHM as a function of the demodulation order is shown in Fig. S11f, and is in the range 128-175 nm; this can be considered as an estimate of the spatial resolution, Δx. These values correspond to ~ λ/600 and ~λ/800 of the impinging wavelength, respectively, indicating a strong light confinement. The spatial resolution increases with the demodulation order due to the tip-sharpening effect.

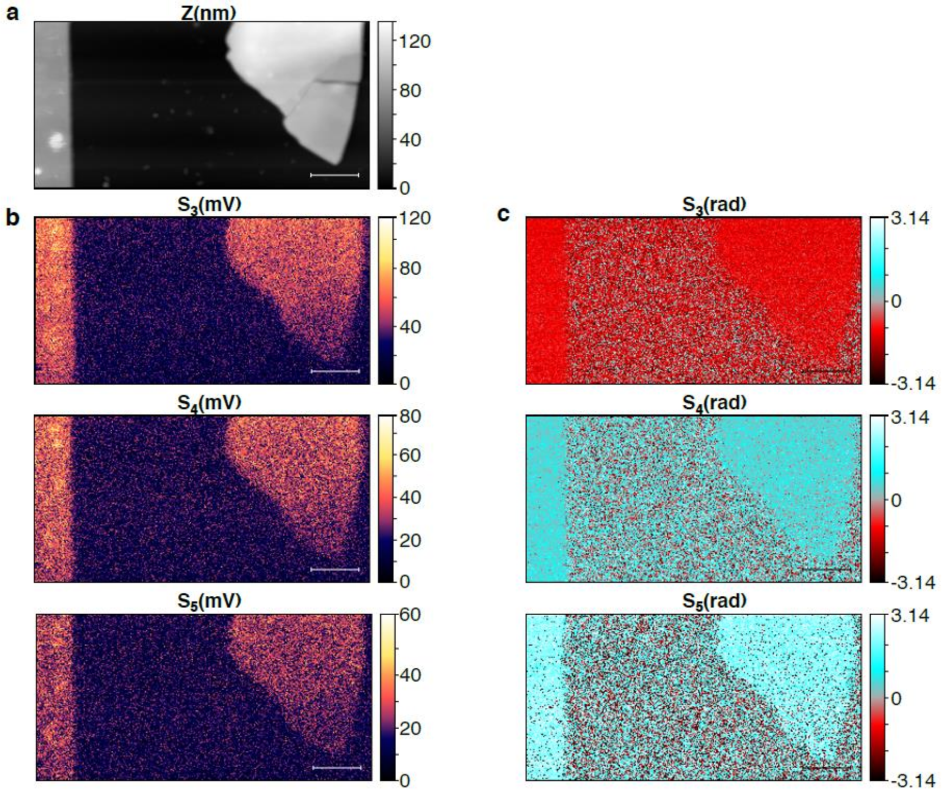


**Figure S12: Near-field imaging with $FC_1$ of a $Bi_2Se_3$ flake. a,** AFM map of the topography of an Au marker (on the left) and a $Bi_2Se_3$ flake (on the right) separated by undoped $SiO_2$/Si substrate. The scale bar corresponds to a length of 2 μm. **b,** Near-field amplitude maps corresponding to the topographical map in (a) obtained from $FC_1$ for the demodulation orders n = 3, 4, 5. **c,** Near-field phase maps corresponding to the topographical map in (a) obtained from $FC_1$ for the demodulation orders n = 3, 4, 5.

We then mapped, with steps of 46 nm, a large area (14 μm x 7 μm) of the sample, shown in Fig. S12a, comprising a gold marker (left) and a 100 nm thick triangular flake of $Bi_2Se_3$ (right). The near-field self-mixing maps acquired, while keeping the optical path length fixed, are reported in Fig. S12b-c. Interestingly, even though we are using a tip with 4 times smaller apex radius, the retrieved signal is much more intense than observed with $FC_2$ in the multi-beatnote regime. This indicates a stable operating condition, against thermal and mechanical drifts, which is ideal for measurements requiring long acquisition time. The higher signal is related to the superior coherence state of $FC_1$ at this bias point, as testified by the observed single and sharp beatnote. The high signal provides a good contrast up to the 5th harmonics, see bottom panels of Fig. S12b-12c. The SNR at n=3 is equal to 5.6 with 7 ms

integration time, compared with a value 4.6 for $s_3$ achieved with a 100ms per pixel integration time for the map in Fig. 4b with $FC_2$. However, $FC_2$ was intentionally driven at the bias point corresponding to the broadest spectrum to guarantee the highest spectral coverage for the hyperspectral imaging.

The similar signal amplitude from the gold marker and the $Bi_2Se_3$ flake is seen from their comparable reflectivity around 3.25 THz, in agreement with the results achieved with $FC_2$.

### 10. Spatial resolution of near-field imaging experiment with $FC_2$

The spatial resolution of the hyperspectral near-field experiment with $FC_2$, and a 40 nm AFM tip, was established by analysing the near-field signal jump at the interface of an Au marker of 45 nm thickness. The near-field self-mixing signal for demodulation orders n = 2, 3, 4 is reported in Fig. S13a-b.

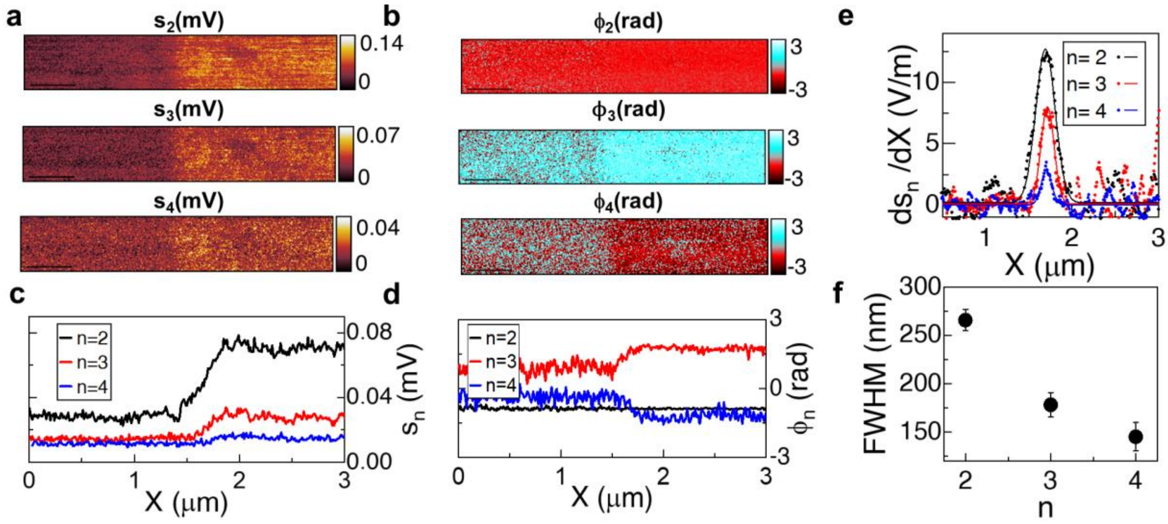


**Figure S13: Near-field imaging spatial resolution using $FC_2$. a,** Amplitude of the near-field self-mixing signal $s_n$ obtained by mapping a 45 nm-thick gold marker on a Si substrate for demodulation orders n=2, 3, 4. $FC_2$ was driven at 300mA, and the AFM tip had a 40 nm apex radius. **b,** Phase, $\phi_n$, of the near-field self-mixing signal corresponding to $s_n$ in (a) for demodulation orders n = 2, 3, 4. **c-d,** Line-profiles extracted as cross-sectional average of the maps in (a-b) for (c) the signal amplitude, $s_n$, and (d) the phase, $\phi_n$. **e,** Line-spread function (LSF) obtained by evaluating the first space-derivative of the line profiles of the signal amplitudes in (c) (colored dots), together with the Gaussian fit (solid colored line) for demodulation orders n=2, 3, 4. **f,** FWHM of the LSF of (e), extracted from the Gaussian fit which can be assumed to be an estimate of the spatial resolution of our near-field imaging system.

We plot the line profiles in Fig. S13c-d from the cross-section average of the maps in Fig. S13a-d, and then use the first derivative of the signal amplitudes to estimate the LSF of our system (Fig. S13e). The FWHM obtained by analysing the LSF curves with a Gaussian fit is

reported in Fig. S13f. This is taken to be an estimate of the spatial resolution of our imaging system, and is found for n=3 to be ~175 nm, corresponding to ~ λ/570 of the THz wavelength.

### 11. Spectral phase of the self-mixing signal

Figure S14 shows the spectral map of the phase, $\phi_3$, of the self-mixing signal of $FC_2$, retrieved while scanning the sample along the direction crossing the substrate and the $Bi_2Se_3$ flake (red dashed line, Fig. 4a main text). The map is built by performing a Fourier transform analysis of the data of Fig. 4e (main text) as a function of time delay.

As the counterpart to the power spectrum in Fig. 4f, Fig. S14 shows the frequency of modes in the range 2.9-3.5 THz that contribute most significantly to the self-mixing signal. It also shows that these FC modes conserve their mutual phase relationship throughout the scan over the topographically-flat $Bi_2Se_3$ flake.

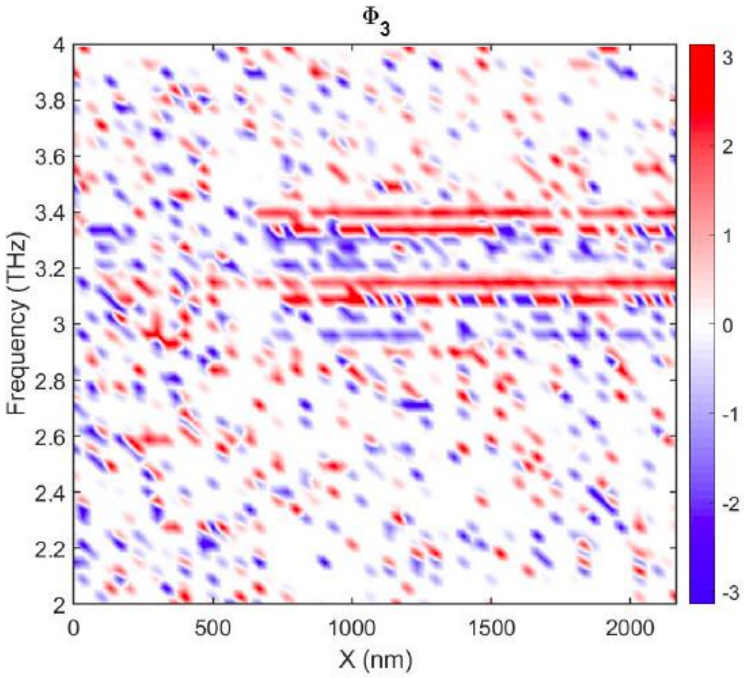


**Figure S14**: **Spectral phase map of $s_3$.** The data, expressed in radians, is obtained by moving along the trajectory, X, that passes from the substrate to the $Bi_2Se_3$ flake, and performing a Fourier transform of the data of Fig. 4e as a function of time delay; this figure complements the power spectrum of Fig. 4f.

### 12. Near-field response of $Bi_2Se_3$ and Au

Figures S15a-n show the X and Y component of the self-mixing signal recorded on Au (Fig. S15a-f) and $Bi_2Se_3$ (Fig. S15g-n) as a function of the demodulation order (n=1, 2, 3).

It can be seen that the signal intensity decreases with increasing demodulation order as the far-field components of the back-scattered radiation are removed. From the quadrature, s, of the X and Y components, one can readily retrieve the spectral dependence of the signal amplitude for Au and $Bi_2Se_3$, respectively (Fig. 4g, main text, for n=3).

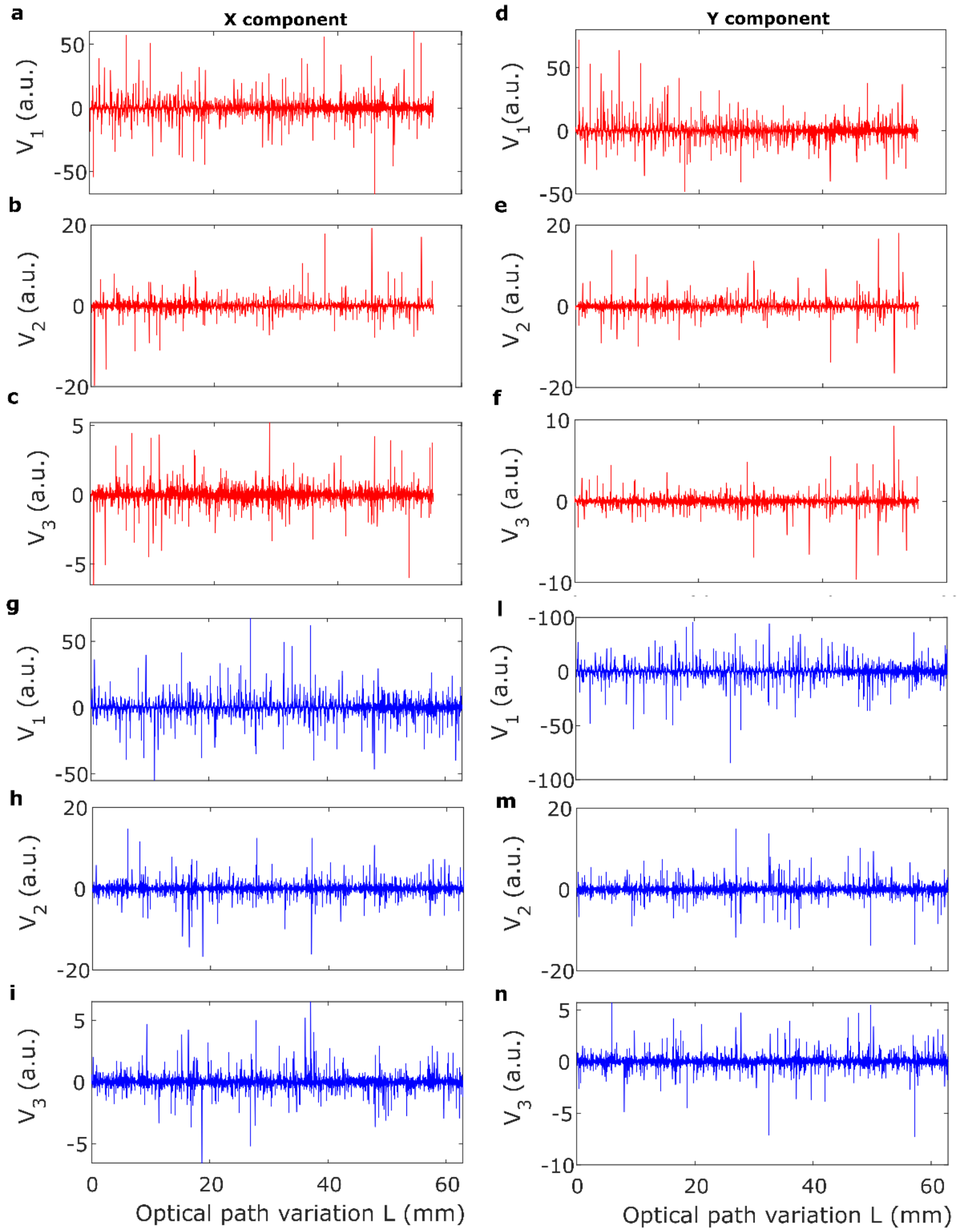


**Figure S15: Near-field self-mixing signal $V_n$ time traces. a-n,** Signals acquired on Au (a-f) and $Bi_2Se_3$ (g-n) separated into X (left panels, a-c and g-i) and Y (panels on the right, d-f, l-n) components at demodulation orders n=1, 2, 3, and acquired while driving $FC_2$ at 480 mA. From the signal amplitude $s=\sqrt{X^2+Y^2}$, we calculate the spectra presented in Fig. 4g (main text).

## 13. THz-TDS hyperspectral nanoimaging

The performances of the THz near-field microscope based on THz-QCL frequency combs are compared with that of state-of-the-art THz hyperspectral near-field microscopes[7] based on a broadband time-domain spectroscopy system (TDS). The setup for THz-TDS hyperspectral nanoimaging measurements includes an InAs photoconductive antenna which, pumped by a 1560 nm femtosecond laser (Menlo System), emits a THz pulse, whose spectrum is extended from 0.3-5THz. The emitted THz pulse is then focused on the AFM tip by the parabolic mirror of the s-SNOM. The portion of the THz radiation which is forward-scattered by the tip is collected by the parabolic mirror and detected by a second photoconductive antenna, operated in reverse mode, which is used as receiver. The near-field component of the signal is isolated through the lock-in detection of the components oscillating at the harmonics of the tip tapping frequency. The waveform of the THz of pulse is reconstructed by measuring the electric field as a function of time, changing the delay time between emission and detection with optical delay line. Third-order near-field signal maps of the $Bi_2Se_3$ flake of Fig.4, measured with the TDS-THz system are reported in Fig. S16 as obtained using the same tip used for $FC_2$, operated in tapping mode at 22kHz with 170nm tapping amplitude. The third order signal amplitude ($s_3$) and phase ($\varphi_3$) collected on the flake are acquired keeping fixed the time delay, which is chosen as the maximum of electric field amplitude. The waveform of the near-field scattered THz pulse is then measured keeping fixed the position on the sample and performing a 30 ps time scan, integrating 200ms per time-delay. The curves obtained by averaging 10 different time scans, on the $Bi_2Se_3$ flake and on gold, are reported in Fig. S17. The near-field spectra, reported in Fig. 4g, are then retrieved by performing the Fourier transform analysis of the time traces in Fig. S17.

Due to the low optical power and low scattering efficiency of the AFM tip for frequencies >2 THz, a SNR>1 is obtained only in the range 0.4 - 1.8 THz (blue line in Fig.4h) which is thus complementary with respect to the frequency range that we can access with the near-field nanoscope based on THz-QCL FC developed in the present work, as demonstrated through the near-field spectra collected with $FC_2$ in the 2.0-3.6 THz range.

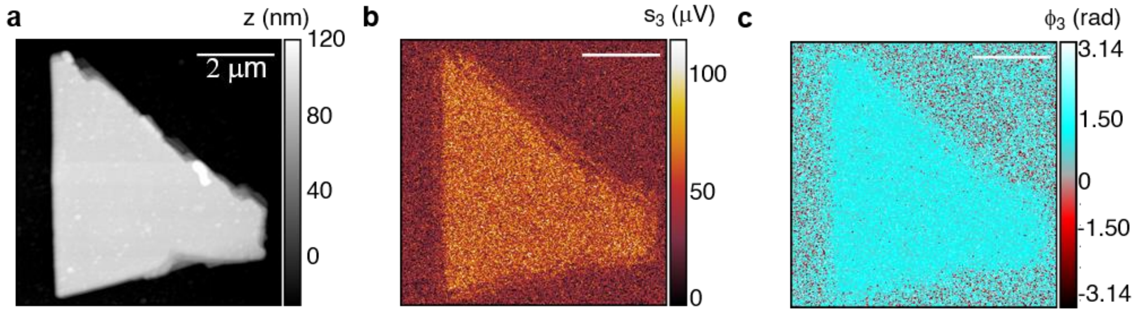


**Figure S16: Hyperspectral nanoimaging with THz-TDS**: **a**, topography of the $Bi_2Se_3$ flake of Fig. 4; **b-c,** third-order near-field signal amplitude $s_3$ (b) and phase $\phi_3$(c) acquired with TDS-s-SNOM system[9] based on InAs photoconductive antennas at fixed time delay *t* with nitrogen purging to suppress absorption from water vapour (humidity <4%).

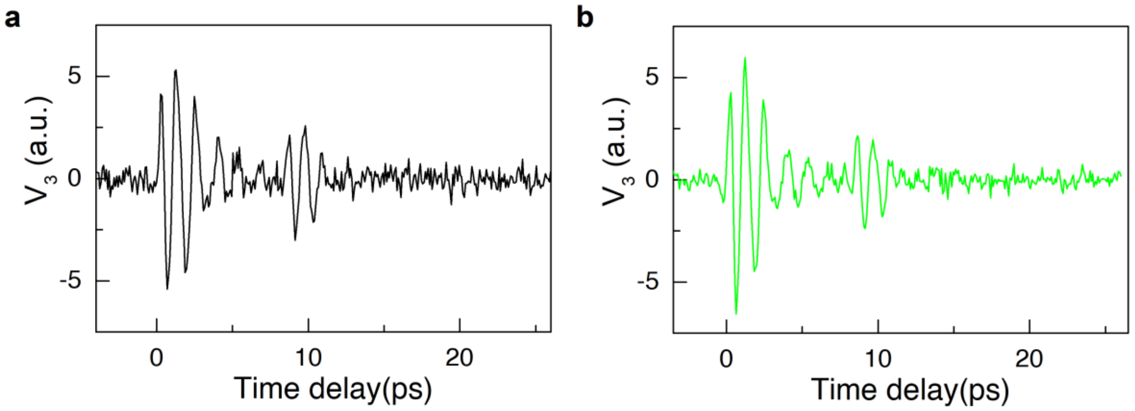


**Figure S17: Near-field THz-TDS time traces a-b,** Third-order near-field signal $V_3$ as a function of the time delay, obtained integrating 200ms per time delay, measured on a fixed position on the gold marker (a) and on the $Bi_2Se_3$ flake(b).

## 14. Noise equivalent power estimation

To obtain an estimate of the sensitivity of our phase-sensitive hyper-spectral near-field nanoscope, we followed the procedure described in Ref. 8. We monitored the amplitude variation of the self-mixing signal as a function of the feedback intensity by inserting a double-pass attenuator into the optical path whilst recording the self-mixing signal with $FC_2$ driven at 300mA as the tip approached the 45-nm-thick gold marker. We note that the signal reaches the noise level of our lock-in detection line (0.1μV, mostly limited by the QCL driver) for a filter with 1% transmission, i.e. -40dB attenuation. Considering that the laser at 300mA delivers a power of 4mW into the external cavity, this would correspond to an NEP of 400 pW/√Hz. This represents a high estimate of the NEP, since with a tip of larger radius or using a larger tapping amplitude, we can easily achieve a smaller NEP. Based on a recent

study[9], an enhancement of one order of magnitude is indeed expected by changing the tip radius from 25 to 750 nm.

In Figure S18, we show the near-field self-mixing signal from FC1 overwhelmingly improved when the source is operating as a comb at 440 mA, compared to the case in which the QCL operates as a generic multimode source. The spectrum of the two time traces (panel c) reveal that the spectral information is accessible only when the QCL's modes are phase-locked, corroborating our conclusion that phase-locking is the fundamental condition to perform near-field hyperspectral imaging with Fabry-Perot THz QCLs.

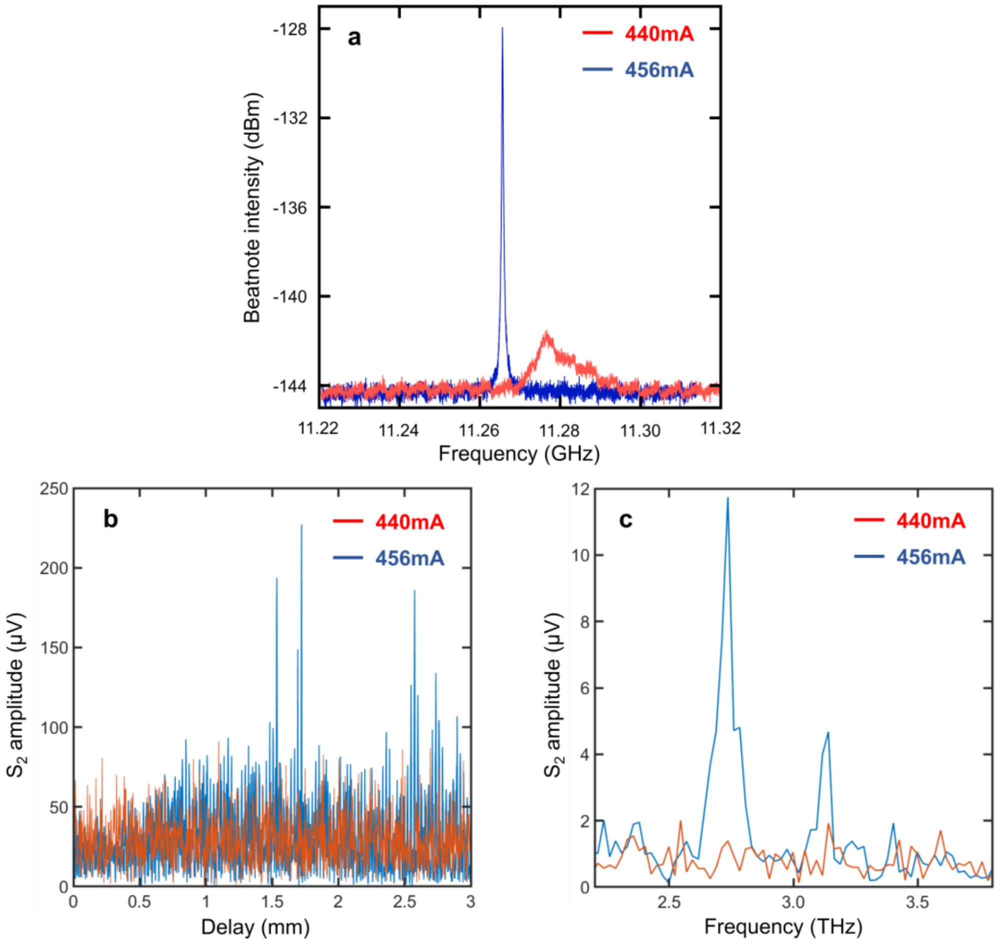


**Figure S18:** **a**, Beatnote spectra of FC1 at 440 mA (blue curve) and 456 mA (red curve), corresponding to a narrow and strong beatnote, and a broad and weak beatnote, respectively. **b,** Near-field signal demodulated at the second harmonics of the tapping frequency of the tip ($S_2$) recorded by moving the delay line over 3 mm, i.e. about one QCL cavity round-trip, while the QCL is coupled to the AFM tip of the s-SNOM, approached to a Au marker. **c,** Fourier components of the second harmonics near-field self-mixing signal at 440 mA and 456 mA.

## References


1. F. P. Mezzapesa, L. L. Columbo, M. Brambilla, M. Dabbicco, M. S. Vitiello, G. Scamarcio *Appl. Phys. Lett.* **2014**, *104*, 041112.
2. E. A. A. Pogna, C. Silvestri, L. L. Columbo, M. Brambilla, G. Scamarcio, M. S. Vitiello *APL Photonics* **2021**, *6*, 061302.
3. A. Forrer, M. Rösch, M. Singleton, M. Beck, J. Faist, G. Scalari *Opt. Express* **2018**, *26*, 23167.
4. A. Forrer, Y. Wang, M. Beck, A. Belyanin, J. Faist, G. Scalari *Appl. Phys. Lett.* **2021**, *118*, 13111.
5. J. Zhang, Z. Peng, A. Soni, Y. Zhao, Y. Xiong, B. Peng, J. Wang, M. S. Dresselhaus, Q. Xiong *Nano Lett.* **2011**, *11*, 2407.
6. K. Garrasi, F. P. Mezzapesa, L. Salemi, L. Li, L. Consolino, S. Bartalini, P. De Natale, A. G. Davies, E. H. Linfield, M. S. Vitiello *ACS Photonics* **2019**, *6*, 73.
7. N. A. Aghamiri, F. Huth, A. J. Huber, A. Fali, R. Hillenbrand, Y. Abate *Opt. Express.* **2019**, *27*, 24231.
8. J. Keeley, K. Bertling, P. L. Rubino, Y. L. Lim, T. Taimre, X. Qi, I. Kundu, L. H. Li, D. Indjin, A. D. Rakić, E. H. Linfield, A. G. Davies, J. Cunningham, P. Dean *Opt. Lett.* **2019**, *44*, 3314.
9. C. Maissen, S. Chen, E. Nikulina, A. Govyadinov, R. Hillenbrand *ACS Photonics* **2019**, *6*, 1279.
